# Multifunctional Chiral Halide Perovskites: Advancing Chiro‐Optics, Chiro‐Optoelectronics, and Spintronics

**DOI:** 10.1002/advs.202509155

**Published:** 2025-07-12

**Authors:** Qi Liu, Hui Ren, Qi Wei, Mingjie Li

**Affiliations:** ^1^ Department of Applied Physics The Hong Kong Polytechnic University Hung Hom Kowloon Hong Kong 999077 China; ^2^ Shenzhen Research Institute The Hong Kong Polytechnic University Shenzhen Guangdong 518057 China; ^3^ Photonics Research Institute The Hong Kong Polytechnic University Hung Hom Kowloon Hong Kong 518057 China

**Keywords:** chiral halide perovskite, chiral optoelectronic devices, chirality optimization, chirality transfer, nanoscale fabrication, spintronics

## Abstract

Chiral halide perovskites (CHPs) represent a revolutionary material class, integrating the exceptional optoelectronic properties of halide perovskites with chirality. This unique combination enables advanced functionalities in chiroptics, spintronics, and next‐generation optoelectronics. Recent breakthroughs highlight CHPs’ capabilities in circularly polarized light (CPL) emission/detection, spin‐selective charge transport, and nonlinear optical processes, establishing them as a focal point in multifunctional material research. This review provides an in‐depth, device‐centric analysis of the latest CHP technologies. Material design strategies, chirality induction/transfer mechanisms, scalable synthesis methods, and diverse device architectures are explored. Particular emphasis is placed on clarifying structure–property–performance relationships across applications, including CPL photodetectors, light‐emitting diodes, lasers, second‐harmonic generation devices, spintronic components, and neuromorphic optoelectronics. Additionally, CHPs’ potential for cutting‐edge applications such as multimodal polarimetry, artificial intelligence, and secure information processing is examined. By defining design guidelines and performance benchmarks, this review aims to bridge the gap between academic research and practical technology translation. It not only synthesizes the current state‐of‐the‐art but also outlines future directions for high‐performance CHP devices, driving progress in this rapidly evolving field.

## Graphical abstract

This review spotlights key advances in chiral halide perovskite materials and devices, covering device architectures, fabrication methods, and emerging applications like circularly polarized light functions, neuromorphic computing, and spintronics. It explores structure‐property relationships and chirality transfer mechanisms, discusses challenges in material stability and scalable manufacturing, and envisions interdisciplinary integration for future optoelectronic and quantum technology growth.

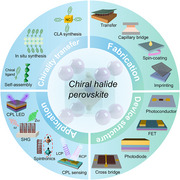

## 1 Introduction

Chirality (from the Greek “cheir”, meaning “hand”) refers to an intrinsic geometric property of an object that possesses non‐superimposable mirror‐image forms. Such objects lack an internal plane of symmetry and cannot be spatially aligned with their mirror counterparts through rotations or translations, thus existing exclusively as two enantiomorphic states: left‐handedness and right‐handedness.^[^ ^1^, ^2^, ^3^ ^]^ An object that conforms to the chirality definition exhibits mutually opposite chiral configurations to its mirror image.^[^ ^4^, ^5^ ^]^ This phenomenon is ubiquitously observed across natural and artificial materials, spanning scales from macroscopic systems (e.g., galactic spiral arms, and planetary rotation) to microscopic entities (e.g., amino acids, deoxyribonucleic acid, organic polymers, nanomaterials, and metasurface).^[^ ^6^, ^7^, ^8^, ^9^ ^]^ Notably, chirality gives rise to a spectrum of unique photophysical phenomena, including nonlinear optical (NLO) responses, chirality‐induced spin selectivity (CISS), circular dichroism (CD), circularly polarized photoluminescence (CP‐PL), and spin‐polarized exciton dynamics. These chirality‐driven effects unlock revolutionary opportunities in quantum optoelectronics, spintronic devices, and chiral biosensing.^[^ ^10^, ^11^, ^12^, ^13^ ^]^

Over the past decade, nanostructured halide perovskites (defined by crystal structure or morphology definition) have been a focal point in optoelectronic research due to their superior properties and intriguing features.^[^ ^14^, ^15^, ^16^ ^]^ For example, 2D (n = 1) and quasi‐2D (n > 1) Ruddlesden‐Popper (RP) phase halide perovskites (A′_2_A_n‐1_B_n_X_3n+1_, where A′ is monovalent cation, A is organic cation, B is divalent metal cation and X is halide anion) show precisely tunable bandgap, layer‐engineered quantum confinement as well as enhanced anti‐humidity stability.^[^ ^17^, ^18^, ^19^ ^]^ For RP phase halide perovskites, the inorganic metal‐halide layers (ε_inorganic_ ≈ 7.3) are spatially confined by wide‐bandgap organic spacers with low dielectric constants (ε_organic_ ≈ 4), mimicking quantum well (QW) structures.^[^ ^20^ ^]^ This dielectric contrast creates strong exciton confinement, yielding large exciton binding energies (>100 meV), reduced exciton radii (≈2–4 nm), and prolonged radiative lifetimes. Moreover, the organic ligands further dictate structural rigidity, interlayer spacing, and moisture resistance, empowering precise modulation of properties.^[^ ^21^, ^22^, ^23^ ^]^ Harnessing these exceptional attributes, halide perovskites show groundbreaking performance across diverse optoelectronic applications, including light‐emitting diodes (LEDs), photovoltaics, photodetectors (PDs), lasers, and neuromorphic photonic synapses.^[^ ^24^, ^25^, ^26^, ^27^, ^28^, ^29^ ^]^ Nevertheless, their intrinsic centrosymmetric crystal symmetry inherently contradicts chirality, precluding their utilization in chiroptical applications such as circularly polarized light (CPL) sensing, CPL emission, and spin‐polarized carrier filter, a critical limitation that recent advances in symmetry‐breaking strategies aim to overcome.

The pursuit of chirality in halide perovskites has spurred four primary engineering strategies, including the incorporation of chiral cations (direct synthesis and surface modification with chiral ligands), ion doping, chiral template growth, and hybridization with chiral metasurfaces.^[^ ^30^, ^31^, ^32^, ^33^, ^34^ ^]^ Among these, embedding chiral ligands into halide perovskite disrupts their centrosymmetric frameworks, accomplishing chirality transfer from molecular to lattice levels. This synergy yields chiral halide perovskites (CHPs) that infuse multiple excellent properties of perovskites with the specific chirality of chiral molecules.^[^ ^35^, ^36^, ^37^ ^]^ The dynamic interplay between chiral ligands and halide perovskite matrices unlocks a wealth of unprecedented optoelectronic functionalities. On the one hand (optic perspective), CHPs exhibit giant CD, implementing polarization‐selective photon harvesting. Concurrently, their non‐centrosymmetric lattices facilitate efficient second‐harmonic generation (SHG), and spin‐polarized exciton transport and CP‐PL emerge from chirality‐mediated spin–photon coupling. On the other hand (electric perspective), the chiral ligand framework induces CISS, steering spin‐polarized currents with near‐unity efficiency, and enhanced spin‐orbit coupling (SOC) and ferroelectric polarization enable electric‐field‐tunable spin manipulation. This combination of optical and spintronic traits positions CHPs as versatile platforms for next‐generation chiroptoelectronics containing polarization‐resolved PDs, 3D displays, spin‐filtering logic gates, and non‐volatile memory.^[^ ^38^, ^39^, ^40^ ^]^ Compared to conventional halide perovskites, current CHPs‐based research remains nascent, and guidelines for CHPs‐based devices are obscure. Critical gaps persist in many aspects, from understanding the chirality transfer mechanism to device‐scale integration, which discounts the development of CHPs in cutting‐edge technologies, including non‐volatile memory, CPL sensing, circularly polarized LEDs, bio‐imaging, and quantum computing.

**Figure 1:**
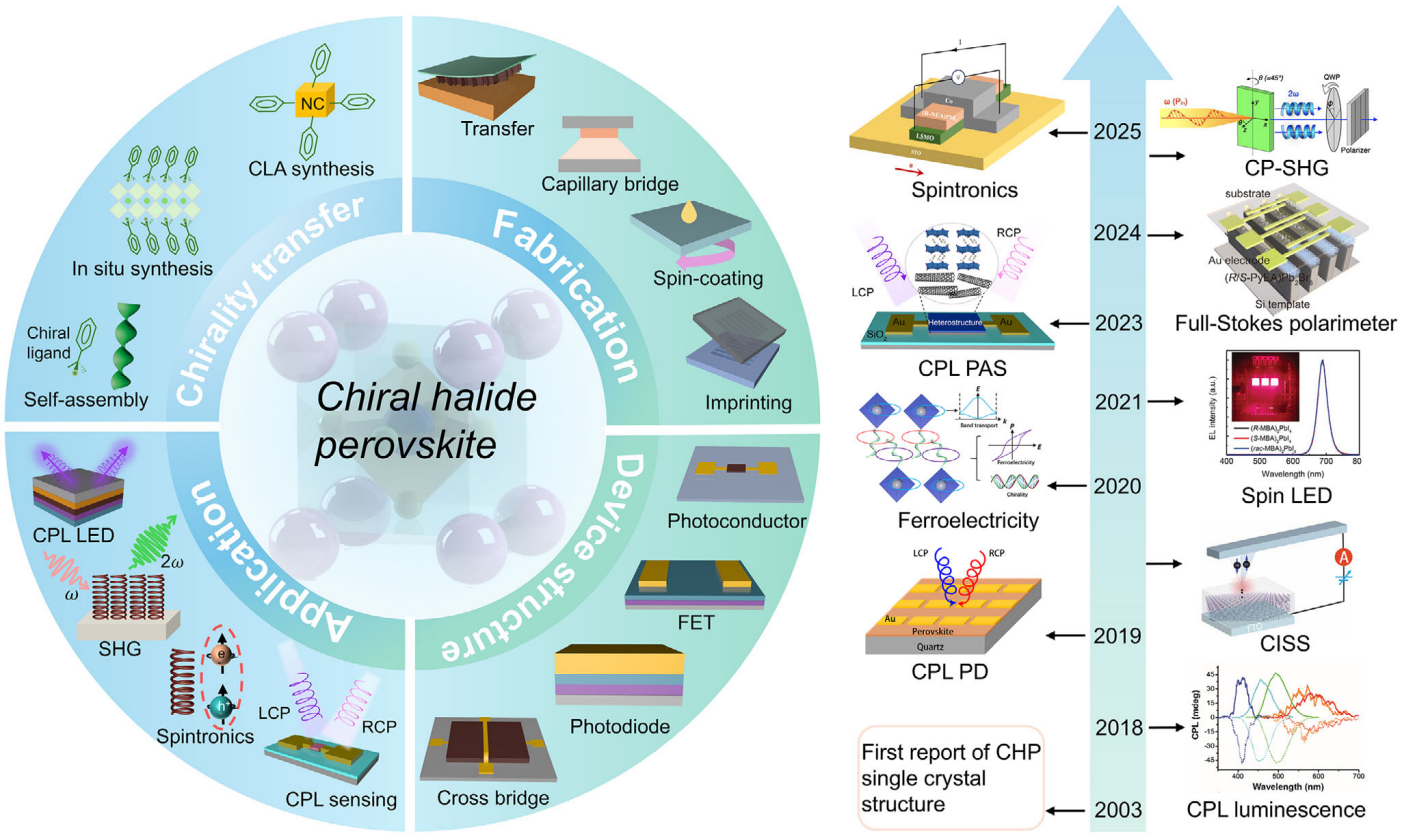
Schematic diagram (left) of chirality transfer, fabrication process, device design, and advanced applications for CHPs. Historical progression of innovative CHPs‐based studies (right). Reproduced with permission.^[^ ^41^ ^]^ Copyright 2018, Wiley‐VCH. Reproduced with permission.^[^ ^42^ ^]^ Copyright 2019, Nature Publishing Group. Reproduced with permission.^[^ ^43^ ^]^ Copyright 2019, AAAS. Reproduced with permission.^[^ ^44^ ^]^ Copyright 2020, AAAS. Reproduced with permission.^[^ ^45^ ^]^ Copyright 2021, AAAS. Reproduced with permission.^[^ ^46^ ^]^ Copyright 2023, Nature Publishing Group. Reproduced with permission.^[^ ^47^ ^]^ Copyright 2024, American Chemical Society. Reproduced with permission.^[^ ^48^ ^]^ Copyright 2024, Wiley‐VCH. Reproduced with permission.^[^ ^49^ ^]^ Copyright 2025, American Chemical Society.

Building upon the abovementioned challenges, this review pioneers a systematic exploration of CHPs in interdisciplinary frontiers from a device‐centric perspective. The integrated framework spanning chirality transfer mechanisms, micro/nanofabrication, device engineering, and functional applications is shown in **Figure** **1**. In Section 2 of this work, we describe the chirality transfer mechanism of CHPs and highlight the mainstream synthesis methods, including chiral‐ligand‐assist (CLA) method, in situ synthesis, and direct mixture, clarifying the coupling rules between molecular chirality and the perovskite crystal lattice and band structure across multiple scales. Moreover, a series of strategies for chirality optimization of CHPs are analyzed. In Section 3, emerging fabrication processes developed for CHPs with various morphologies are presented. In detail, the transfer technique for thin‐layer CHP crystal, capillary bridge effect for CHP nanowires (NWs), spin‐coating method for film, and template‐based imprinting for microwires (MWs) are introduced. In Section 4, we analyze architectures, carrier transport, interface effects, and operation rules of various CHP‐based devices, including two‐terminal photoconductors and photodiodes, three‐terminal field‐effect transistors (FETs), and four‐terminal cross‐bridge devices. In Section 5, the advanced applications covering CPL sensing photodetector (PD), photonic artificial synapse (PAS), CPL LEDs, chiral lasing, spintronics based on the CISS effect, and nonvolatile memory, which are achieved or expected on CHPs, are recommended. Ultimately, the current issues and challenges in developing CHP‐based devices with various applicability are shared, and suggestions for constructing next‐generation multifunctional chiral‐optoelectronics and spintronics are listed in terms of the present status of devices built upon CHPs. These insights aim to provide theoretical support and a technological roadmap for developing high‐performance and miniaturized chiral optoelectronic devices, propelling CHPs from fundamental research toward the implementation of functionalized system integration.

## 2 Chirality of CHPs

Chirality transfer describes the phenomenon where chiral molecules instill their handedness into an object's structure through various mechanisms: chemical bonding, spatial arrangement, and electronic interactions. This process profoundly influences the optical, electrical, and spin properties of the resulting material.^[^ ^50^, ^51^, ^52^ ^]^ Advancing fundamental understanding of CHPs hinges on a comprehensive exploration of their structures, chirality transfer mechanisms, synthesis methods, and strategies for optimizing chiral properties. These interconnected research directions will not only illuminate the structure‐property relationships within CHP systems but also provide a framework for rationally designing and precisely engineering CHPs with tailored optoelectronic properties, ultimately propelling innovation in this exciting field.

### 2.1 Structure and Chirality Transfer

**Figure 2:**
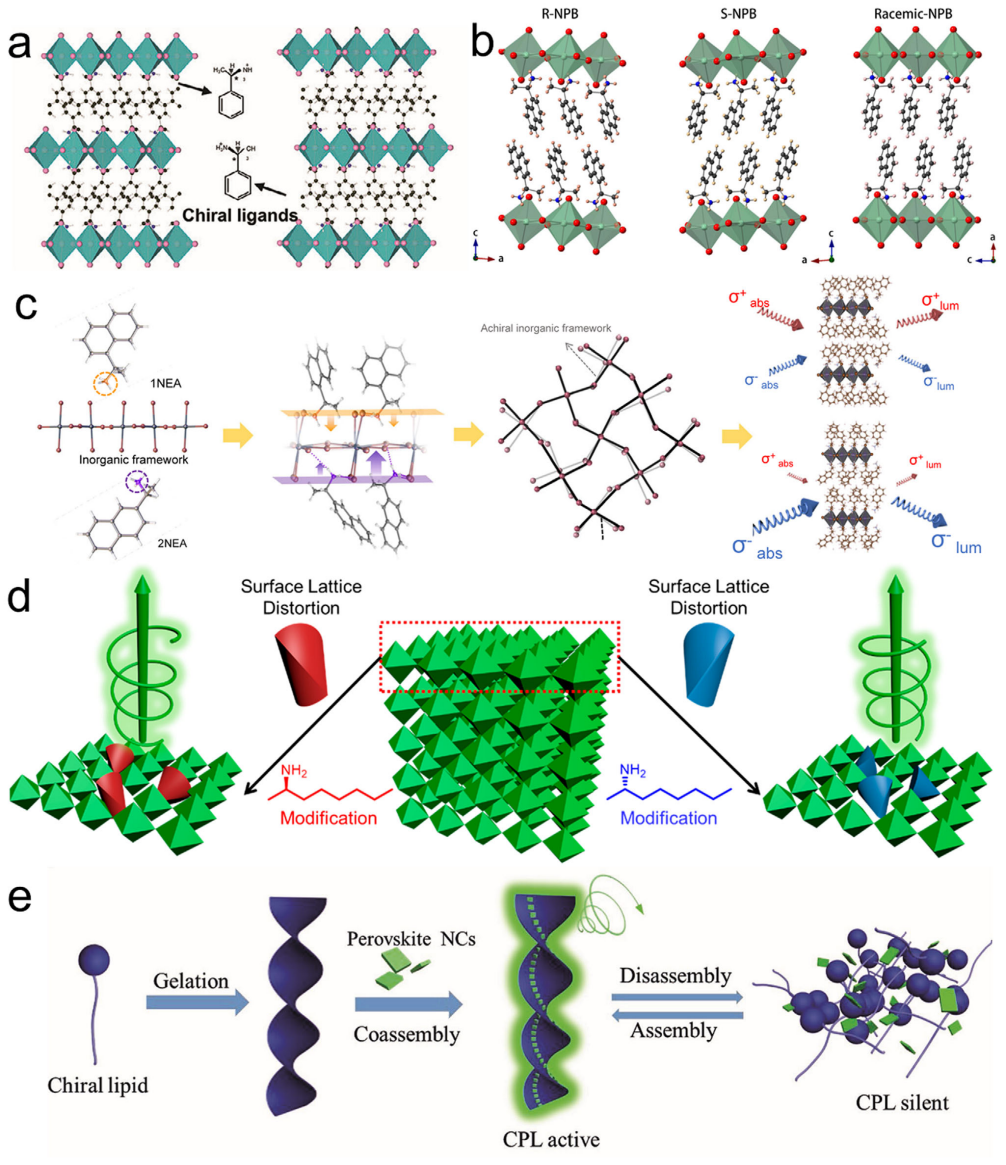
Typical chirality transfer mechanism. a) Crystal structures of 2D (R‐MBA)_2_PbI_4_ (left) and (S‐MBA)_2_PbI_4_ (right). Reproduced with permission.^[^ ^61^ ^]^ Copyright 2025, Wiley‐VCH. b) Single‐crystal structures of 1D R‐type (left), S‐type (middle), and racemic (right) CHP. Reproduced with permission.^[^ ^62^ ^]^ Copyright 2020, Nature Publishing Group. c) Stepwise schematic for illuminating the chirality transfer mechanism of CHPs including the diverse molecular structure, hydrogen interaction, chirality transferred to achiral inorganic framework, and induced chiroptical response. Reproduced with permission.^[^ ^63^ ^]^ Copyright 2019, American Chemical Society. d) Chiral ligand‐induced surface lattice distortion of halide perovskite NCs. Reproduced with permission.^[^ ^64^ ^]^ e) chiral self‐assembly of perovskite NCs. Reproduced with permission.^[^ ^41^ ^]^ Copyright 2018 Wiley‐VCH.

3D chiral metal halide perovskites possess a general chemical formula of ABX₃, where A represents a monovalent cation, B denotes a metallic cation, and X signifies a halide anion. These materials feature corner‐sharing BX_6_ octahedra forming a 3D framework, with A cations residing within the cavities of this framework.^[^ ^39^, ^53^ ^]^ Crucially, the incorporation of smaller A cations into these cavities is essential for achieving a stable structure in 3D CHPs. Quasi‐2D and 2D CHPs share structural similarities with the RP perovskite, adopting a chemical formula of (RNH_3_)_2_A_n‐1_BX_3n+1,_ where n = 1 designates a 2D structure, while n > 1 represents a quasi‐2D arrangement. In these structures, RNH_3_ signifies the chiral ligand situated between the inorganic layers.^[^ ^54^, ^55^ ^]^ As shown in **Figure** **2**a, enantiomers *R*‐ and *S*‐α‐Methylbenzylamine (R‐MBA and S‐MBA, MBA = C_6_H_5_C_2_H_4_NH_2_) with different handedness could serve as the organic part for synthesizing 2D CHPs (n = 1, B is Pb, X is I) (R‐MBA)_2_PbI_4_ and (S‐MBA)_2_PbI_4_. Resembling the component tunability of the RP perovskite, CHPs with 2D and quasi‐2D dimensionality can be fabricated flexibly by tuning the value of *n* from 1 to ∞, This strategy effectively improves the charge and energy transfer in chiroptical devices. The first reported 1D CHPs, with the general formula (RNH_3_) BX₃, emerged in 2003. These materials result from utilizing chiral cations and metallic cations in a 1:1 molar ratio.^[^ ^56^ ^]^ Figure 2b draws the 1D crystal structure of R‐type, S‐type, and racemic CHPs (R ligand: S ligand = 1:1), in which distorted face‐sharing BX_6_ octahedral chains are encompassed by a chiral packing framework. Bridging the dimensional gap between 2D and 1D chiral perovskites, a special 1.5D structure, (R‐MBA)(GA)PbI₄, was first introduced by Xiao et al. in 2025.^[^ ^57^ ^]^ The synergistic effect of GA and R/S‐MBA components leads to the formation of a robust hydrogen‐bonding network. This network orchestrates the well‐ordered arrangement of zigzag 1D chains, effectively interweaving them within layered structures that bridge the gap between traditional 1D chains and 2D layer CHP architectures. The dimensionality classification of CHPs, ranging from quasi‐2D to 1D, is fundamentally rooted in the connectivity patterns of octahedra, which represent the building blocks at the molecular level. However, a distinct perspective on 1D CHPs emerges when considering morphology. When two dimensions of the CHP material shrink to the nanoscale range, nanowire (NW) structured 1D CHPs arise.^[^ ^58^ ^]^ When all three dimensions of a CHP are reduced to the nanoscale range, 0D CHPs emerge, manifesting as quantum dots (QDs) or nanocrystals (NCs).^[^ ^59^, ^60^ ^]^ Unlike their bulk film counterparts, these low‐dimensional morphologies exhibit a significantly enhanced surface‐to‐volume ratio. Coupled with strong quantum confinement effects, these characteristics synergize with the inherent properties of CHPs, unlocking a vast potential for constructing advanced nanoscale chiral‐optoelectronics and spintronics.

Unraveling the chirality transfer mechanism serves an important role in the guidance of synthesizing high‐quality CHP for remarkable optoelectronics. Recent studies have proposed several chirality transfer mechanisms in halide perovskite: I) chiral ligand‐induced chiral structure; II) chiral ligand‐induced distortion (chiral footprint) or dislocations; III) electronic coupling between chiral and achiral building blocks; IV) chiral self‐assembly.^[^ ^65^ ^]^ Within CHP crystals, chirality can be effectively transferred from organic molecules to the halide perovskite framework by substituting conventional organic cations with chiral ligands. This substitution disrupts the inversion symmetry of the crystal structure, inducing a chiral arrangement within the inorganic framework of PbX_6_ octahedra. Notably, this incorporation does not compromise the advantageous properties of the inorganic component, such as high carrier mobility and excellent absorption/emission characteristics, which compensate for any potential limitations in electrical conductivity posed by the chiral organic molecule. In 2023, Moon et al. illuminated the plausible stepwise chirality transfer process of 2D CHPs, which is based on crystallographic studies and chiroptical spectroscopy, and corresponding details of the analysis are pictured in Figure 2c.^[^ ^63^ ^]^ The enantiomeric molecular configurations enhance hydrogen‐bonding interactions between the chiral cations and the host inorganic lattice, inducing lattice distortions in the inorganic (PbBr_6_)^4−^ frameworks and facilitating chirality transfer from the organic isomers to the inorganic matrix. This structural asymmetry subsequently activates chiroptical responses at the first excitonic absorption edge, driven by spin‐split excitonic transitions within the distorted inorganic sublattice.

Perovskite NCs can obtain chirality through ligand‐induced surface lattice distortion. The binding of chiral ligands not only imparts stereochemical asymmetry but also modulates the electronic properties of NCs via synergistic structural distortion and interfacial orbital interactions between organic ligands and the inorganic [PbX_6_]^4−^ octahedral framework. For instance, Duan et al. verified that chiral ligand‐functionalized CsPbBr_3_ NCs exhibit intense CPL emission behavior, attributed to ligand‐mediated chirality transfer (Figure 2d).^[^ ^64^ ^]^ In this system, chiral ligands selectively anchor to bromide‐rich surface defects, distorting the NCs’ atomic arrangement and breaking local symmetry, thereby activating pronounced chiroptical responses. Beyond intrinsic chirality from crystalline asymmetry, supramolecular chiral self‐assembly offers a macroscopic strategy to endow halide perovskites with emergent chirality. Liu et al. reported that achiral perovskite NCs co‐assembled with chiral gelators acquire macroscopic handedness through interfacial chirality transfer (Figure 2e).^[^ ^41^ ^]^ The gelators adhere to NC surfaces, inducing helical alignment of the hybrid framework and generating CPL with a dissymmetry factor (g_lum_) of ≈10⁻^3^. This supramolecular approach bypasses complex chemical synthesis, which is conductive to the scalable fabrication of flexible CPL devices with tunable polarization states.

### 2.2 Synthesis Methods of CHPs

**Figure 3:**
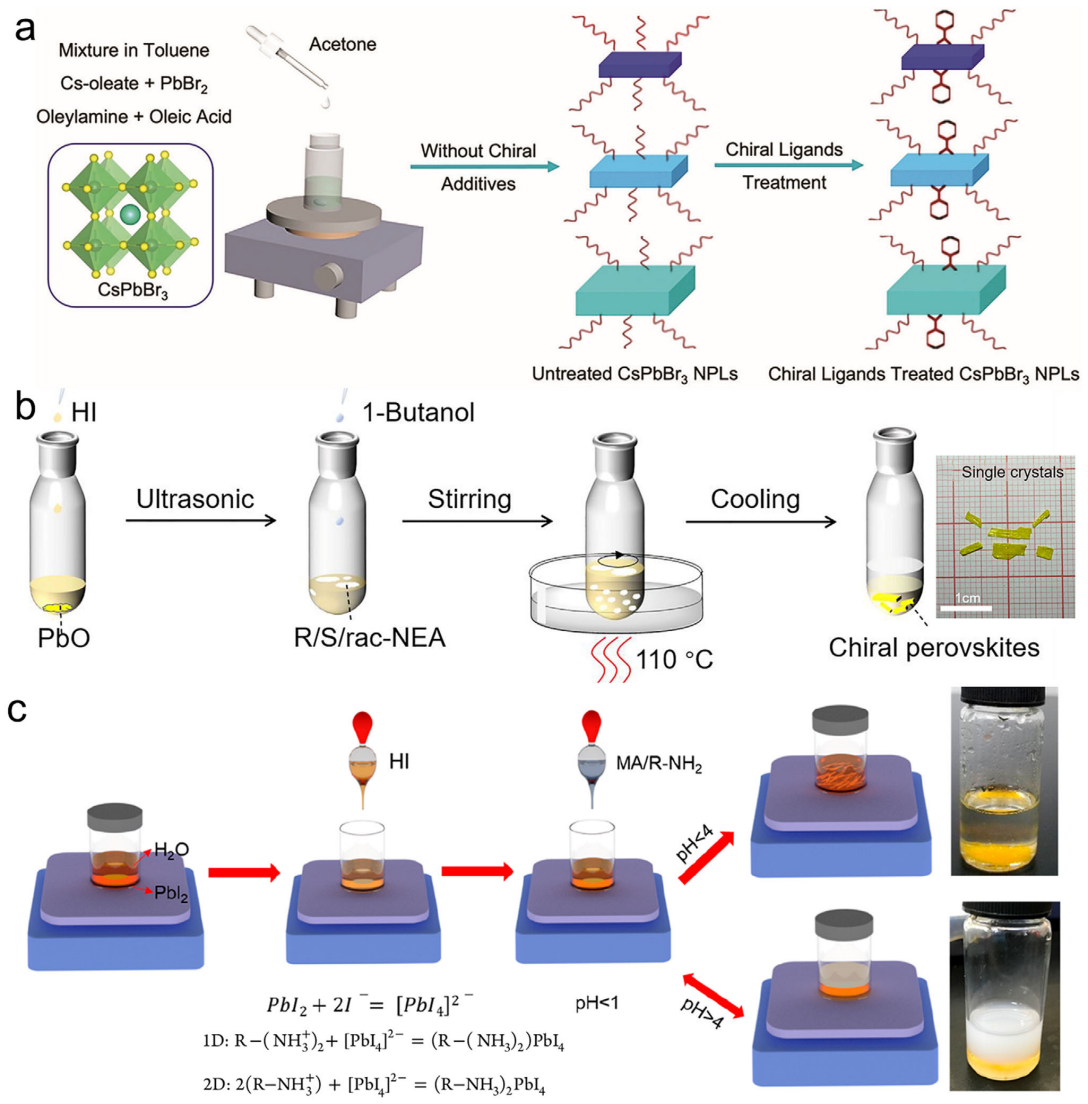
Synthesis method for CHPs. a) Preparation of chiral CsPbBr_3_ nanoplates capped with R‐/S‐PEABr ligands. Reproduced with permission.^[^ ^66^ ^]^ Copyright 2024, Wiley‐VCH. b) Cooling crystallization method for synthesizing (S/R‐NEA)PbI_3_ and (rac‐NEA)_2_PbI_4_. Reproduced with permission.^[^ ^67^ ^]^ Copyright 2022, American Chemical Society. c) Aqueous synthesis method with different solution pH to prepare 1D and 2D CHPs, respectively. Reproduced with permission.^[^ ^68^ ^]^ Copyright 2019, American Chemical Society.

Chirality transfer in CHPs is directly governed by their synthesis methodologies, which can be categorized into two dominant approaches based on the sequence of chiral ligand incorporation: chiral‐ligand‐assisted (CLA) post‐synthetic modification and direct in situ chiral synthesis. In the CLA method, pre‐synthesized achiral perovskite nanocrystals are functionalized with chiral ligands through surface coordination, where stereospecific ligand–perovskite interactions drive chirality transfer. A representative example by Rogach et al. demonstrated CLA‐mediated synthesis of chiral CsPbBr_3_ nanoplates (NPLs), where chirality originates from ligand‐induced lattice screw dislocations (**Figure** **3**a).^[^ ^66^ ^]^ The process involves three consecutive stages. Initially, CsPbBr_3_ NPLs are synthesized at room temperature via an anti‐solvent method, where acetone triggers the crystallization of Cs‐oleate and PbBr₂ precursors. Subsequently, a post‐synthetic ligand exchange is performed using R‐ or S‐PEABr enantiomers under sonication to impart chiral functionality to the NPLs. Finally, centrifugal purification is employed to isolate the chirality‐active NPLs, removing unreacted ligands and byproducts to achieve a high‐purity colloidal dispersion. The stereoselective binding of PEABr ligands to bromine‐terminated surface sites amplifies lattice distortion, creating helical dislocation networks that couple with quantum‐confined excitons. This synergy endows the NPLs with tunable chiroptical responses, including exciton energy splitting and circularly polarized emission, providing a blueprint for designing CHPs with programmable spin‐photon interactions.

Direct synthesis of CHPs permits dimensional control (0D–3D) by integrating chiral ligands with perovskite precursors during crystallization under precisely tuned conditions. Widely adopted techniques, i.e., antisolvent crystallization, cooling crystallization, aqueous synthesis, and evaporation crystallization, offer versatile routes to engineer CHP architectures. For example, Tian et al. synthesized 1D chiral (R/S‐NEA)PbI_3_ single crystals via cooling crystallization (Figure 3b).^[^ ^67^ ^]^ The protocol involves dissolving PbO in hydroiodic acid within a sealed reactor, followed by controlled addition of chiral NEA ligands. After stirring the precursor at 110 °C to form a homogeneous yellow solution, slow cooling at programmed rates induced asymmetric crystallization, yielding millimeter‐scale CHP crystals. Alternatively, Li et al. developed an aqueous‐phase synthesis for low‐dimensional CHPs by leveraging pH‐dependent coordination chemistry (Figure 3c).^[^ ^68^ ^]^ Initially, PbI_2_ exhibits limited solubility in deionized water but forms soluble [PbI_4_]^2−^ complexes upon HI addition. Subsequent injection of methylammonium (MA) and chiral PEA ligands triggers dimensional control: acidic conditions (pH < 2) favor 1D C_4_N_2_H_14_PbI_4_ chains, while neutral pH directs 2D (S/R‐PEA)_2_PbI_4_ layered structures. This eco‐friendly aqueous approach achieves ≈80% yield, double that of conventional cooling crystallization, by minimizing ligand desorption and phase segregation, thereby strengthening scalable CHP production for industrial applications.^[^ ^69^ ^]^

### 2.3 Chirality Optimization

The chiroptical properties of CHPs, including CD, circularly polarized luminescence, and spin polarization, are pivotal for their applications in spin‐resolved photonics and quantum optoelectronics. Enhancing these chirality‐dependent properties is essential to improve the performance of CHP‐based devices. To optimize the chirality, a series of strategies including doping,^[^ ^70^ ^]^ chemical composition engineering,^[^ ^71^ ^]^ solvent‐mediated crystallization,^[^ ^72^ ^]^ and dimensional confinement have been proposed.^[^ ^73^, ^74^ ^]^ For example, targeted doping with specific cations or anions strengthens CD signals by modulating lattice symmetry and spin‐orbit interactions. Solvent engineering fine‐tunes crystallization kinetics to stabilize chiral phases with reduced defects, while dimensional control in 2D/3D heterostructures enhances CPL intensity via quantum‐confined exciton‐photon coupling. Additionally, chiral ligand passivation suppresses non‐radiative losses to prolong spin‐polarized carrier lifetimes. These coordinated efforts collectively address the critical link between chirality enhancement and device performance, and bring breakthroughs in spin‐polarized LEDs, polarization‐discriminative PDs, and high‐density spintronic memory systems.

**Figure 4:**
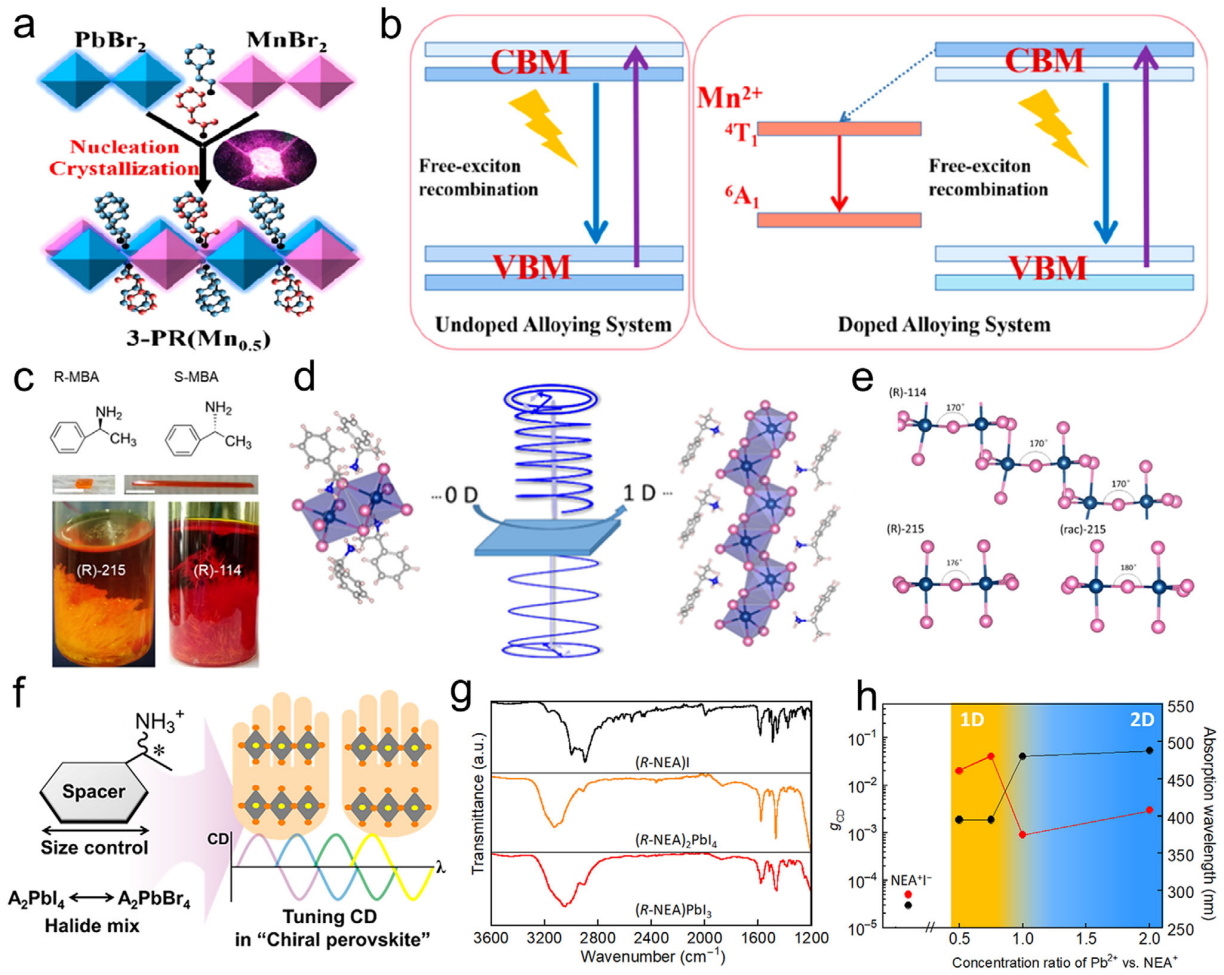
Chirality optimization methods. a) Structure diagram of Mn‐doping CHP. b) Energy transfer and enhanced PL mechanism of 2D CHP doped with Mn^2+^. Reproduced with permission.^[^ ^75^ ^]^ Copyright 2021, American Chemical Society. c) Molecular structures of chiral ligands (up), chiral perovskite crystals (middle), and as‐grown 0D (R/S‐MBA)_2_SbI_5_ and 1D (R/S‐MBA)SbI_4_ (down), namely (R)‐215 and (R)‐114. d) Schematic illumination of dimension‐enhanced chirality. e) Inorganic framework of 0D, 1D, and racemic CHP. Reproduced with permission.^[^ ^73^ ^]^ Copyright 2022, American Chemical Society. f) Chemical composition modification for tuning CD signals. Reproduced with permission.^[^ ^78^ ^]^ Copyright 2020, American Chemical Society. g) Fourier transform infrared (FTIR) spectra of (R‐NEA)I, (R‐NEA)_2_PbI_4_, and (R‐NEA)PbI_3_. h) The g_CD_ factor and absorption corresponding to Pb^2+^: NEA^+^ ratio. Reproduced with permission.^[^ ^81^ ^]^ Copyright 2020, AAAS.

Zhao et al. doped manganese ions (Mn^2+^) into 2D CHP in which chiral cations and achiral inorganic blocking co‐exist, and obtained efficient photoluminescence (PL).^[^ ^75^ ^]^ As illustrated in **Figure** **4**a, the crystal structure comprises blue [PbBr_6_]^4−^ and pink [MnBr_6_]^4−^ octahedra, with the inset showing bright emission under 365 nm UV excitation. To illuminate the enhancement of the PL, the author sketches the bandgap and energy transfer process. Under excitation, the sample is excited from the ground state to the free exciton state, followed by a radiation recombination process (free exciton return to the ground state). The doping of Mn^2+^ provides a new radiation path, namely the exciton recombination way, where the energy is transferred from the free exciton to the ^4^T_1_ state of manganese ions. This energy transfer of light‐stimulated exciton from the host to the inside Mn^2+^ facilitates the radiation recombination, thereby boosting the PL performance.

Similarly, Lu et al. achieved CP‐PL and exciton splitting in Mn^2+^‐doped (S/R‐MPA)_2_PbBr_4_, where increasing Mn^2+^ concentration boosted the PL quantum yield to 24% and dissymmetry factor to 0.11 (Figure 4b).^[^ ^70^ ^]^ The enhancement aligns with Zhao's energy transfer model, confirming the universality of Mn^2+^‐mediated chirality amplification. Expanding this strategy, Co^2+^ doping in achiral perovskites was shown to induce a Zeeman effect, breaking spin degeneracy and generating spin‐polarized carrier populations.^[^ ^76^ ^]^ This spin imbalance enhances CP‐PL and photoresponse, achieving a 15% improvement in spin polarization efficiency compared to undoped systems. Collectively, these studies validate ion doping as a versatile strategy to engineer chiral activity through tailored spin‐photon interactions, offering a scalable route to high‐performance chiral‐optoelectronics.

The dimensional engineering of CHPs offers a powerful route to amplify chirality. Tang et al. systematically compared 0D (R)‐215 and 1D (R)‐114 CHPs (Figure 4c), revealing a tenfold enhancement in CD anisotropy factor of (R)‐114 (Figure 4d).^[^ ^73^ ^]^ Density functional theory (DFT) calculation elucidates that the frontier orbitals of conduction band minimum (CBM) and valence band maximum (VBM) of (R)‐114 and (R)‐215 are composed of inorganic sublattices with antimony and lead‐derived states, and the optimized chirality may come from the chiral‐ligand‐improved inorganic configuration. Further, the bending angles of the octahedrons in 1D ([Sb_2_I_8_]_n_^2n‐^ loop chain) and 0D ([Sb_2_I_10_]^4‐^ dimer) are sketched to understand the structure‐induced CD difference. The former exhibits a higher bending angle than the latter, while the racemic perovskite shows no bending angle, and the author deduced that this structural difference from dimensionality might interpret the CD strength. Complementing this, Choi and co‐workers achieved dimensionality‐tunable CHPs using three distinct chlorine‐substituted ligands.^[^ ^77^ ^]^ The substitution position of the chlorine atom decides the structural dimensionality and chiroptical properties of CHP. When the chlorine‐substituted ligands were shifted to the ortho position, 1D CHP with self‐powered sensing mode exhibited the best anisotropy factor of 1.25. The work manifests how dimensionality control, guided by substituent positioning, allows CHPs to surpass classical chiroptical limits, opening avenues for self‐powered polarization sensors and chiral photonic circuits.

Chemical composition engineering, particularly through halide alloying and cation substitution, serves as a potent strategy to amplify chirality in halide perovskites by directly modulating their electronic and stereochemical landscapes. Moon et al. validated this by tuning the halide composition in (R/S‐MBA)_2_PbI_4(1−x)_Br_4x_, where increasing the Br^−^/I^−^ ratio (x = 0–0.3) blue‐shifted the CD peak from 495 to 474 nm (Figure 4f).^[^ ^78^ ^]^ This shift correlates with Br‐mediated bandgap widening and excitonic state redistribution. Beyond x = 0.3, CD signals vanished due to disrupted long‐range chiral order, whereas substituting MBA with bulkier NEA ligands restored chirality via enhanced magnetic transition dipole moments, further blue‐shifting CD to 375 nm. Extending this approach, Ning et al. reported 2D CHP (R/S‐MBA)_2_Pb_1‐x_Sn_x_I_4_ with different lead‐tin ratios for a high dissymmetry factor CPL detector.^[^ ^79^ ^]^ With the increasing ratio of x, the CD peak around 500 nm gradually weakens (x = 0.2) and disappears (x = 0.5). Ultimately, the CD peak shows a blue shift (x >0.8), mirroring Moon's halide‐dependent trends. However, compositional mixing risks undermining chirality‐driven functionalities. Dan et al. found that the mixed halide in (R/S‐MBA)_2_PbI_4(1−x)_Br_4x_ weakens the CISS effect.^[^ ^80^ ^]^ The mixed CHP showed suppression of chirality during the probe of CD, which was ascribed to the photoinduced halide segregation. The magnetic conductive atomic force microscopy (mc‐AFM) measurements of the CISS behavior showed a decrease upon the introduction of bromide, which could be interpreted by the inhomogeneous strength of the hydrogen bond between the organic groups and halogens in the inorganic framework. Further, the hydrogen bond variation induced the transfer of chirality to the inorganic sublattice from the amine. This compositional sensitivity underscores the delicate balance between bandgap engineering and chiral integrity, necessitating precise doping protocols.

Miyasaka et al. pioneered ultrahigh CD in CHP films (>3000 mdeg) by engineering organic–inorganic interactions in 1D and 2D architectures.^[^ ^81^ ^]^ In the FTIR (Figure 4g), the N‐H stretch band emerges at 3120 and 3050 cm^−1^ for 1D and 2D CHPs, respectively, which indicates the hydrogen ion of −NH_3_^+^ group in NEA^+^ intensively interacts with the iodide ion in (PbI_6_)^4−^. It can be seen that the 1D CHP shows a stronger interaction indicated by the peak intensity. Further, the relationship between the dimension of CHP and chiroptical properties is explored and plotted in Figure 4h, and the CHP exhibits a higher anisotropy factor of CD (g_CD_, calculated by CD/(32 980 × absorbance)) in 1D structure. In addition to the strong bonding interaction, another key factor is the addition of methylammonium iodide (MAI) in the synthesis process of (R/S‐NEA)PbI_3_. The MAPbI_3_ coexists with the 1D phase CHP in the film. Under the synergistic effect of the MAPbI_3_ and (R/S‐NEA)PbI_3_, the fabricated film exhibits an extremely high CD signal, about an order of magnitude higher than that without the addition of MAI. The crystallinity and the surface smoothness of the 1D CHP film are dominated by the coexistence of MAPbI_3_. The CHPs without the addition of MAPbI_3_ exhibit amorphous states, which significantly discount the chiroptical properties. This work underscores dimensional control and phase engineering as dual levers for maximizing chirality in hybrid perovskites.

While existing optimization strategies effectively modulate the chirality and chiroptical properties of CHPs, critical gaps persist compared to the mature fabrication for achiral perovskites. A central challenge lies in the reliance on chiral ligands to induce asymmetry, which often triggers racemization or uneven spatial distribution of chiral centers during synthesis, compromising chiral purity. Precise control of the arrangement of chiral molecules or lattice distortion should be paid much more attention. The modulation effect of CHPs can be directly observed from CD spectra or luminescence, but the corresponding chirality enhancement mechanism is obscure. Current optimization approaches also suffer from methodological monotony, predominantly focusing on compositional or dimensional tuning, with scant exploration of multifield coupling strategies (e.g., magnetic‐light‐electric field synergies). For instance, metasurface integration, a proven enabler of photonic breakthroughs in lasers, imaging, and memory, has yet to be harnessed for amplifying chirality in CHPs.^[^ ^82^, ^83^ ^]^ Addressing these limitations demands interdisciplinary innovation, merging chirality‐resolved spectroscopy, ab initio simulations, and heterogeneous integration techniques to unlock CHP potential in multiple application fields (e.g., laser, PDs, 3D imaging, and memory).^[^ ^30^, ^84^ ^]^

### 2.4 Physical Origin of Chirality‐Induced Spin Selectivity

The CISS effect in CHPs stems from the intricate interplay between chiral structural motifs and SOC, establishing a unique mechanism for spin‐selective charge transport. The concept of CISS was first introduced in the field of spintronics research. It was proposed as a novel phenomenon where the chirality of molecular species could impart significant spin selectivity to various electron processes. This concept has since been the subject of extensive research, aiming to understand the underlying mechanisms and explore potential applications in areas such as spintronic devices.^[^ ^85^ ^]^

**Figure 5:**
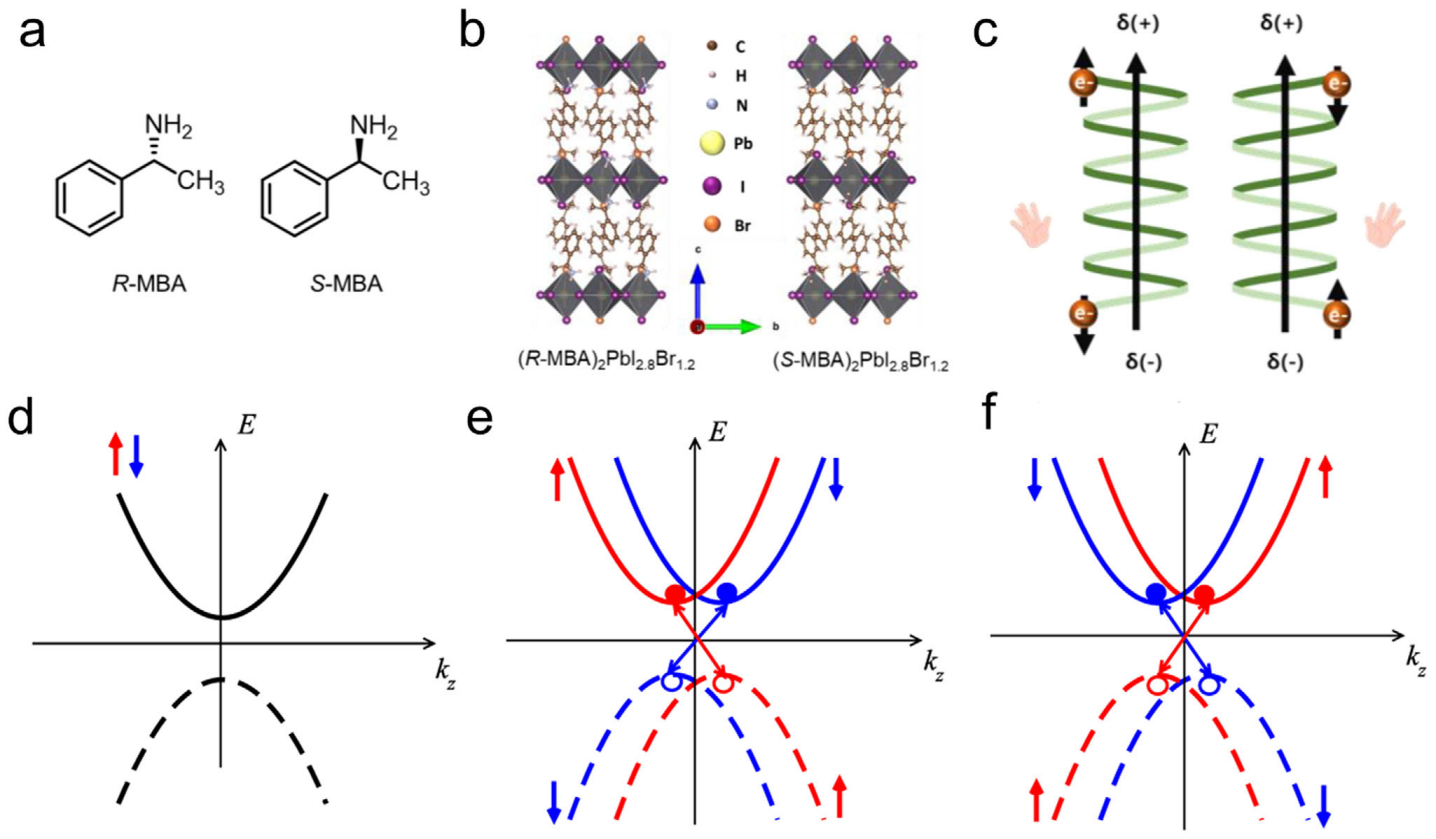
CISS in CHPs. a) R‐type and S‐type chiral ligands. b) Crystal structure of (R‐/S‐MBA)_2_PbI_2.8_Br_1.2_. c) Sketch a diagram of CISS effect. Reproduced with permission.^[^ ^87^ ^]^ Copyright 2024, AIP Publishing. d) Band for achiral perovskite. e) Spin‐polarized sub‐bands split in k‐space with *E* ^+^. f) Spin‐polarized sub‐bands split in k‐space with *E* ^‐^. Reproduced with permission.^[^ ^88^ ^]^ Copyright 2020, American Chemical Society.

In CHPs, the helical arrangement of chiral organic cations (**Figure** **5**a)—such as *R/S*‐α‐methylbenzylamine (*R/S*‐MBA) or 1‐(1‐naphthyl)ethylamine (NEA)—within the inorganic metal halide framework gives rise to an asymmetric electrostatic potential, forming a chiral electric field (${\vec{E}}_{\mathrm{chiral}}$). As an electron traverses this field, its motion induces a relativistic effective magnetic field ($\vec{B}$) in the electron's rest frame, described by the following fundamental relation:

(1) $$\vec{B}=\frac{1}{c^{2}}\vec{v}\times {\vec{E}}_{\mathrm{chiral}}$$

where $\vec{v}$ represents the electron velocity vector, and *c* is the speed of light in a vacuum. This magnetic field breaks the spin degeneracy of electrons, creating an energy splitting that favors the transmission of specific spin states.^[^ ^86^ ^]^ For instance, in (*R/S*‐MBA)_2_Pb_2.8_Br_1.2_ (Figure 5b), the chiral electric field generated by the helical organic cation layers produces a magnetic field that aligns electron spins parallel to the helical axis, enabling preferential transport of spin‐polarized carriers (Figure 5c).^[^ ^87^ ^]^

In achiral halide perovskite, the dispersion relations of electrons and holes preserve both time‐reversal symmetry and spatial inversion symmetry. These symmetries enforce a double degeneracy in the electronic (hole) sub‐bands (Figure 5d).^[^ ^88^ ^]^ The non‐centrosymmetric crystal structure of CHPs disrupts inversion symmetry, thereby enhancing SOC and inducing spin‐dependent band structure modifications. The spin‐orbit coupling term in the Hamiltonian is given by:

(2) $$H_{\mathrm{SO}}=\lambda \vec{\sigma }\left(\vec{p}\times {\vec{E}}_{\mathrm{chiral}}\right)$$

where λ is the spin‐orbit coupling parameter, $\vec{\sigma }=\left(\sigma_{x},\sigma_{y},\sigma_{z}\right)$ are the Pauli matrices acting on the electron spin space, and $\vec{p}$ is the electron momentum vector.^[^ ^89^ ^]^ This interaction results in spin‐split energy sub‐bands, described by the dispersion relation:

(3) $$E^{\pm }\left(k\right)=\frac{\hbar^{2}k^{2}}{2m}\pm \alpha_{\mathrm{eff}}k\;$$

here, ℏ is the reduced Planck's constant, *m* is the effective mass of the electron, $k=|\vec{k}|$ is the magnitude of the electron wave vector, $\alpha_{\mathrm{eff}}=\frac{\Delta E^{\pm }}{2k_{0}}$ is the spin splitting coefficient (with *k*_0_ as a characteristic momentum), and Δ*E* ^±^ denotes the energy difference between the spin‐up (*E* ^+^) in Figure 5e and spin‐down (*E* ^‐^) in Figure 5f sub‐bands at *k* = *k*_0_. In materials like (*R/S*‐NEA)_2_PbBr_4_, this SOC‐induced band splitting enables selective transport of spin‐up or spin‐down electrons, with the preferential spin state dictated by the chiral configuration of the framework.

Chiral organic cations induce pronounced helical deformations in the inorganic lattice—for example, distorting the [PbBr_4_]^2‐^ octahedra in(*R/S*‐NEA)_2_PbBr_4_—leading to asymmetric bond angles (Δ*β*) and lattice strain. This structural distortion enhances SOC and generates a chiral lattice potential that couples strongly with electron spin, giving rise to a spin polarization degree:

(4) $$P_{s}=\frac{I_{+}-I_{-}}{I_{+}+I_{-}}$$

where *I*_+_ and *I*_‐_ are the electrical currents carried by spin‐up and spin‐down electrons, respectively. Notably, lead‐free CHPs such as (*R/S*‐MBA)_2_SnI_4_ exhibit spin polarization degrees exceeding 90%, attributed to the stronger lattice deformation and enhanced SOC from the heavier Sn^2+^ cation versus Pb^2+^ in traditional CHPs.^[^ ^90^ ^]^

In device applications, the CISS effect enables multifaceted spin filtering mechanisms. In spin‐light‐emitting diodes (spin‐LEDs), CHPs function as spin filters, injecting spin‐polarized holes into the emission layer. In spin valves, CHPs sandwiched between ferromagnetic electrodes could exhibit magnetoresistance responses with the response sign reversing upon inversion of the chiral configuration. The CISS effect also suppresses elastic backscattering of electrons, as spin‐flip processes are energetically unfavourable, enhancing charge transport efficiency in spin transistors. This efficiency could be optimized by heterostructure design, such as coupling CHPs with topological insulators to extend spin diffusion lengths.

Theoretical models, including tight‐binding and scattering calculations, reveal that CISS in CHPs is governed by structural parameters (helical pitch, radius) and SOC intensity.^[^ ^91^, ^92^ ^]^ Shorter pitches and larger radii in chiral lattices enhance spin polarization, while heavier metal ions or lattice distortions increase α_eff_. This spin filtering capability, rooted in chiral electric fields, enhanced SOC, and helical lattice deformations, positions CHPs as promising materials for next‐generation spintronic devices, including spin‐based quantum computing and energy‐efficient memory technologies.

## 3 CHP‐Devices Design and Fabrication

CHPs exhibit extraordinary chiroptical and spin‐optical properties beyond the conventional halide perovskite. To leverage their potential for diverse applications, two critical aspects require meticulous attention: device architecture optimization and fabrication process innovation. First, rational device engineering proves fundamental for achieving high‐performance devices employing CHPs as functional layers. Second, advanced manufacturing techniques can significantly broaden the application scope of CHP‐based systems. However, practical implementation faces challenges due to perovskite materials’ inherent limitations in environmental stability and process compatibility when compared with inorganic counterparts like metal oxides, nitrides, and oxide perovskites. A reasonable design should take multiple factors, including bandgap matching (e.g., electron and hole transport layers align energy levels with perovskite to reduce interface barriers), interface passivation (e.g., reducing defect state density through molecular/ionic modification), encapsulation, and process compatibility into consideration.^[^ ^93^, ^94^, ^95^ ^]^ Those optimizations fulfill synergistic enhancement of device performance while addressing material limitations, ultimately facilitating the transition from laboratory prototypes to practical applications.

### 3.1 Architectures of CHP‐Devices

The structural design of CHP‐based devices serves as a foundational determinant of their operational performance and application, governing both fundamental photophysical processes and practical response behavior. By engineering device architectures, researchers can modulate critical operational parameters such as carrier transport trajectories, photon harvesting efficiency, and interfacial contact.^[^ ^96^, ^97^, ^98^ ^]^ Those factors collectively dictate the ultimate photoconversion quantum yield, temporal response characteristics, environmental tolerance, and spectral operating ranges of the resulting devices. Emerging architectural paradigms could be crystallized into four dominant configurations, each exhibiting distinct structure‐response tailored for specific optoelectronic functionalities. Two‐terminal planar architectures, including photoconductors and photodiodes, leverage in‐plane carrier transport mechanisms to achieve polarization‐discriminative photodetection with sub‐nanosecond response times, making them particularly suitable for high‐speed chiroptical sensing applications. Three‐terminal field‐effect transistor (FET) configurations introduce gate‐tunable capabilities, enhancing dynamic modulation of spin‐polarized charge injections. More sophisticated four‐terminal cross‐point architectures employ orthogonal electrode arrangements to measure the magnetoresistance (MR) of CHPs.

**Figure 6:**
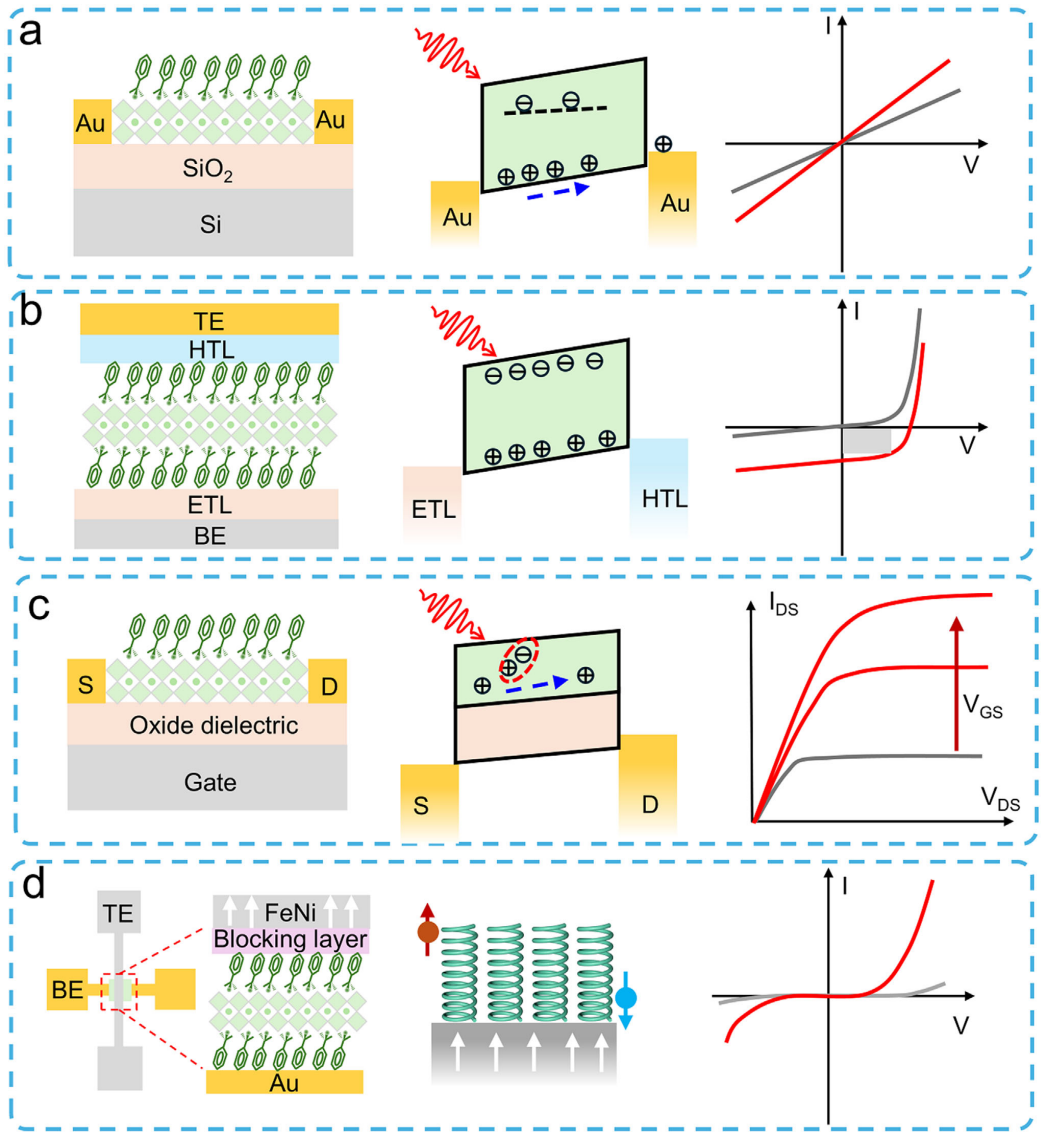
Summarized device structures, carrier transport mechanisms, and *I*–*V* characterization. a) Two‐terminal PD. b) Two‐terminal photodiode. c) Three‐terminal FET. d) Cross‐bridge structure with four terminals.

Photoconductors employ a simple planar architecture where CHP films are directly sandwiched between two metal electrodes (**Figure** **6**a, left). In this configuration, interface contact between the electrode and perovskite critically governs charge transport dynamics. The inherent defect‐rich nature of CHPs introduces a high density of mid‐gap states, which elevates dark current levels but simultaneously accomplishes substantial photoconductive gain. Under an applied electric field, photogenerated carriers undergo multiple transits through the device before recombination, a mechanism where a single photon triggers numerous carrier circulation events, thereby achieving high photoresponsivity. However, these defect states also act as charge traps, prolonging carrier lifetimes and consequently slowing response speeds to microsecond‐millisecond timescales (Figure 6a, middle). Operationally, photoconductors require external bias to sustain Ohmic conduction, with current‐voltage characteristics showing a linear dependence on the applied voltage. Under illumination (red curve), conductivity surges dramatically due to photogenerated carriers, while the baseline dark current (black curve) remains relatively low but exhibits defect‐mediated leakage (Figure 6a, right).

Photodiodes adopt p‐n or p‐i‐n junction architectures (Figure 6b, left), where CHP serves as the photoactive layer and integrates with charge‐selective transport layers. Typically, TiO₂ serves as the electron transport layer (ETL), and Spiro‐OMeTAD is utilized as the hole transport layer (HTL).^[^ ^99^, ^100^, ^101^ ^]^ The built‐in electric field at the heterojunction rapidly separates photogenerated electron‐hole pairs, directing carriers through their respective transport layers to electrodes (Figure 6b, middle). This engineered charge extraction pathway suppresses bulk and interfacial recombination, giving rise to ultrafast nanosecond‐scale response times and almost approaching Shockley‐Queisser efficiency limits. The optimized energy‐level alignment manifests in photovoltaic behavior under illumination, producing measurable short‐circuit currents and finite open‐circuit voltages (Figure 6b, right).

The structure of a CHP FET (Figure 6c, left) closely resembles that of a conventional FET, comprising a semiconductor layer, source (S), drain (D), gate electrode, and dielectric layer.^[^ ^102^, ^103^ ^]^ In this configuration, CHPs act as the conductive channel, where crystal quality critically influences charge transport efficiency and overall device performance. The dielectric layer, typically SiO₂, Al₂O₃, or polymers like PMMA, isolates the gate electrode from the perovskite channel. CHP‐based FETs typically exhibit ambipolar charge transport owing to concurrent electron and hole conduction. Their carrier type dominance (n‐type or p‐type) can be engineered through compositional tuning, such as modifying A‐site cations or adjusting halide ratios. A key advantage of CHPs‐based FETs lies in the strong photon absorption of CHPs, which generates abundant electron‐hole pairs under illumination. An external gate bias facilitates the separation of these photogenerated carriers. Electrons migrate toward the drain electrode, while holes drift to the source, collectively producing a high photocurrent (Figure 6c, middle). The current–voltage (*I*–*V*) characteristics of CHP‐FETs are characterized through transfer curves (ambipolar behavior) and output curves (Figure 6c, right). In the linear regime, the drain‐source current (*I*_DS_) rises linearly with an increasing drain‐source voltage (*V*_DS_), governed primarily by channel resistance. In the saturation regime, *I*_DS_ plateaus and becomes controlled by the gate‐source voltage (*V*_GS_). Through structural design, material optimization (e.g., defect passivation), and interface engineering, CHP‐FETs display significant potential for next‐generation optoelectronic and spin‐selective devices.

When spin‐unpolarized carriers traverse CHPs, they acquire a chirality‐dependent spin polarization via the CISS effect. This property, which controls spin‐filtering charge transport, holds transformative potential for spin‐based memory and spintronic devices. To quantify the CISS effect, a four‐terminal cross‐point device (Figure 6d, left) is employed. The vertical heterostructure comprises an inert bottom electrode (BE;, e.g., Au, Pt), a CHP layer, a blocking layer (e.g., oxide or organic insulator), and a ferromagnetic top electrode (TE;, e.g., Ni, Co, FeNi).^[^ ^104^, ^105^, ^106^, ^107^ ^]^ The MR, defined as the relative change in resistance with magnetization direction, can be obtained using this structure. Under an external sweeping magnetic field perpendicular to the device plane, a constant DC bias is applied between the TE and BE, while the voltage drop across the junction is monitored via the other electrode pair. Unlike mc‐AFM that probes nanoscale regions, this architecture performs macroscopic measurements of spin‐selective transport across bulk CHP films, aligning with the practical behavior of the device.^[^ ^108^, ^109^ ^]^ In this configuration, charge carriers spontaneously develop spin polarization at the termini of the CHP's helical structure (Figure 6d, middle). Spin‐polarized electrons injected from the magnetized ferromagnetic TE propagate through the CHP layer, exhibiting distinct spin‐up and spin‐down currents due to the material's spin‐selective filtering properties (Figure 6d, right). Notably, this four‐terminal design endorses efficient spin injections into nonmagnetic semiconductors or metals without requiring external magnetic fields, a breakthrough for spintronics, such as spin filters, spin‐FETs, and nonvolatile spin memories.

Photodiodes leverage junction‐based built‐in electric fields to achieve efficient charge separation, making them ideal for high‐precision applications such as solar cells and ultrafast PDs. Photoconductors rely on photoconductive gain mechanisms to deliver high sensitivity but face limitations in response speed and long‐term stability due to persistent photocurrent effects. CHPs‐based FETs, with their additional gate terminal, support advanced functionalities by dynamically modulating carrier transport. This architecture not only enhances photodetection performance (e.g., tunable spectral response, on‐chip signal amplification) but also facilitates seamless integration with logic circuits for intelligent optoelectronic systems. Expanding to four‐terminal configurations, the magnetic field modulation is introduced, unlocking novel capabilities. For instance, such designs pave the way for RT high‐efficiency spin‐polarized devices and provide a platform to study emergent multi‐physical coupling (opto‐electric‐magnetic) phenomena in CHP systems. These advances bridge optoelectronics with spintronics, offering tremendous potential for next‐generation memory, sensing, and computing technologies.

### 3.2 Fabrication Techniques of CHPs

**Figure 7:**
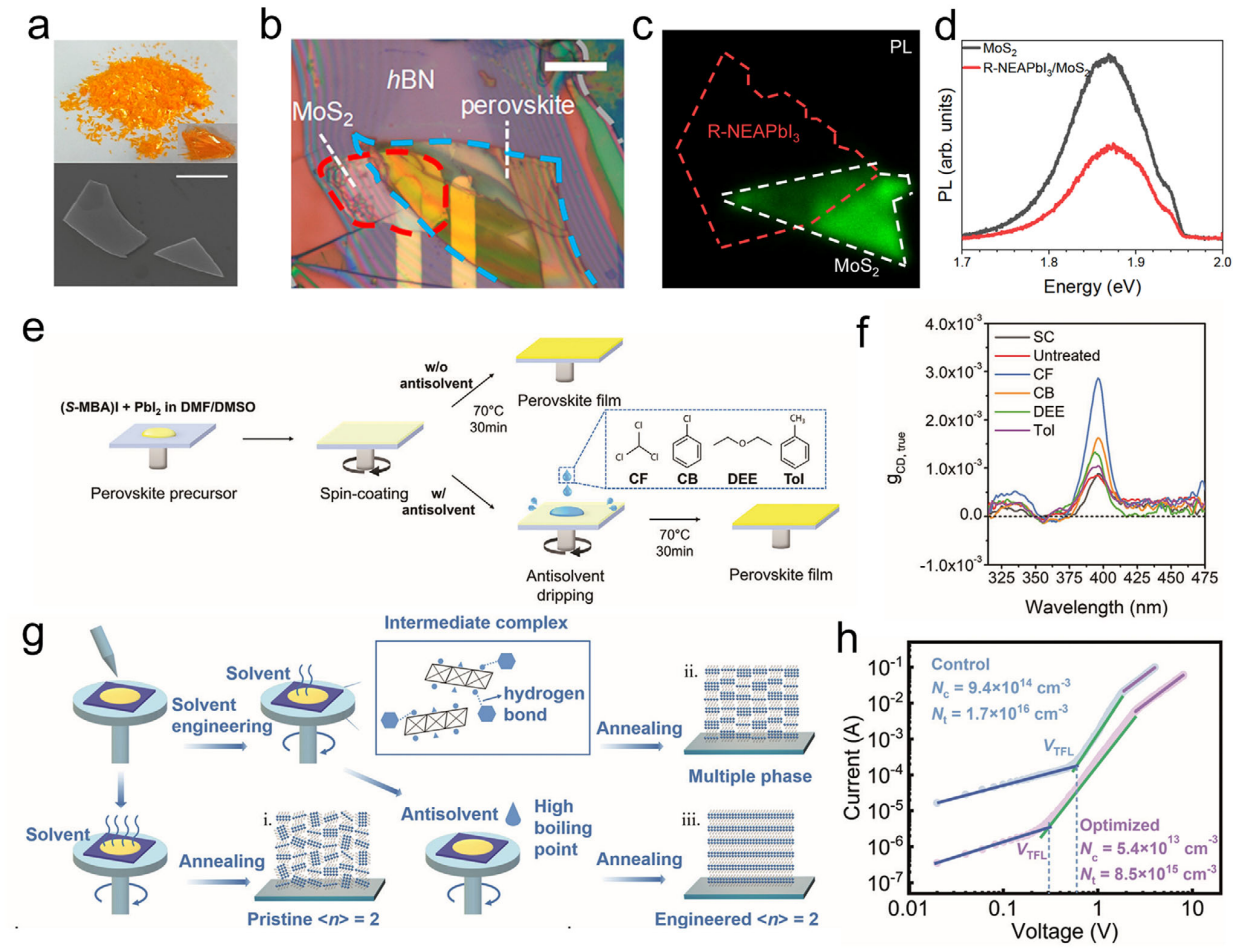
Transfer and spin‐coating methods for CHP‐based devices. a) Optical and SEM images of (R‐ and S‐MBA)_2_PbI_4_ crystal. The scale bar is 5 µm. b). Image of the MoS_2_/CHP heterostructure. Reproduced with permission.^[^ ^113^ ^]^ Copyright 2019, American Chemical Society. c) PL mapping of MoS_2_/R‐NEAPbI_3_. d) PL of MoS_2_/R‐NEAPbI_3_. Reproduced with permission.^[^ ^114^ ^]^ Copyright 2023, Nature Publishing Group. e) Schematic diagram of the fabrication of CHP film. f) Anisotropy factor of CHP by different treatments. Reproduced with permission.^[^ ^72^ ^]^ Copyright 2024, Wiley‐VCH. g) Crystallization mechanism for quasi‐2D CHP film. h) Electrical measurement of pristine and optimized devices. Reproduced with permission.^[^ ^119^ ^]^ Copyright 2020, Wiley‐VCH.

Dry transfer methods using polydimethylsiloxane (PDMS) stamps and wet transfer techniques (e.g., PMMA‐assisted transfer) are widely employed for assembling 2D crystal materials such as graphene and transition metal dichalcogenides.^[^ ^110^, ^111^, ^112^ ^]^ However, transferring CHPs presents unique challenges due to their environmental sensitivity (degradation under humidity/temperature fluctuations) and mechanical fragility. Recent advances in modified transfer methods verify the integration of CHPs with 2D materials, opening avenues for high‐performance optoelectronic devices. In a notable study, Li et al. synthesized millimeter‐scale needle‐like (R/S‐MBA)₂PbI₄ crystals with a distinct orange color (**Figure** **7**a) and constructed a MoS₂/CHP heterostructure to achieve circularly polarized luminescence.^[^ ^113^ ^]^ Exfoliated thin layer of (R‐MBA)_2_PbI_4_ crystal, which was checked by scanning electron microscopy (SEM), exhibited a flat surface owing to the layered properties of 2D CHPs. To fabricate the device, the Au electrode with a channel of 10 µm was defined by photolithography, followed by thermal evaporation and lift‐off. The few‐layer MoS_2_ sheet was aligned and transferred onto one side of Au electrode with the assistance of a microscope and manipulator. The perovskite sheet was exfoliated from the synthesized bulk crystal by Scotch tape, and then was transferred to the other side of the predefined Au electrode. Ultimately, a hexagonal boron nitride (*h‐*BN) flake as an encapsulation layer was transferred onto the top area of the as‐prepared heterostructure to protect it from being damaged by the air (Figure 7b). This CHP/MoS_2_ design achieved circularly polarized luminescence with a dissymmetry factor of 17%, demonstrating significant potential for miniaturized CPL devices. Colet et al. utilized dry transfer methodology to fabricate R/S‐NEAPbI₃/MoS₂ and explored valley polarization inside the heterostructures.^[^ ^114^ ^]^ Monolayer MoS_2_ was prepared utilizing a typical mechanical exfoliation method. Briefly, the few‐layer MoS_2_ bulk on the tape was further folded and peeled several times to thin the flake. The as‐exfoliated monolayer was confirmed by the optical microscope (color), Raman spectroscopy (peak intensity), and atomic force microscope (thickness). For the transfer of the 1D CHP, the R/S‐NEAPbI_3_ crystal was exfoliated on the PDMS stamp, and then the sample was aligned on top of the monolayer MoS_2_ using by transfer stage equipment. The stamp was down slowly and finally contacted the MoS_2_. With the release of the PDMS stamp, the CHP was left on the MoS_2_. Figure 7c exhibits the PL mapping of the R‐NEAPbI_3_/ MoS_2_ heterostructure. The overlap area exhibits an obvious quench signal compared with the MoS_2_‐only region (Figure 7d), indicating a photo‐excited electron transfer from the conduction band of MoS_2_ to R‐NEAPbI_3_. The transfer method in this work demonstrates an innovative way to explore valleytronics devices. Despite these successes, the transfer of CHP remains more challenging than the transfer of 2D transition metal dichalcogenides (TMD) material. Key challenges include susceptibility to lattice distortion during mechanical exfoliation, solvent‐induced degradation in wet transfer processes, and interface contamination from residual polymers (e.g., PDMS/PMMA). Addressing these limitations through advanced encapsulation strategies and solvent‐resistant CHPs is critical for advancing scalable device fabrication.

Spin‐coating, a widely adopted thin‐film deposition technique in perovskite optoelectronics (e.g., solar cells, LEDs, PDs), utilizes centrifugal force to achieve uniform solution distribution.^[^ ^115^, ^116^, ^117^, ^118^ ^]^ The quality of the resulting perovskite film depends on multiple parameters, such as spin speed/duration, solution composition (concentration, solvent selection, and antisolvent treatment), annealing conditions, and ambient environment. Yang et al. enhanced the chiroptical properties of polycrystalline CHP films through antisolvent‐assisted spin‐coating optimization (Figure 7e).^[^ ^72^ ^]^ They synthesized 1D S‐MBAPbI_3_ by dissolving S‐MBAI and PbI₂ in a mixed solution of dimethyl sulfoxide (DMSO)/DMF (30 wt.%). During the spin‐coating process, antisolvents including chloroform (CF), chlorobenzene (CB), diethyl ether (DEE), and toluene (Tol) were dripped to modulate crystallization, followed by thermal annealing at 70 °C for 30 min. The CF‐treated films exhibited the highest *g*_CD_ (Figure 7f), attributed to CF's strong polarity, causing hydrogen bonding with DMSO. This interaction not only suppressed parasitic phase formation but also accelerated 1D perovskite crystallization and reduced iodine vacancy density. The resultant structural homogeneity enhanced asymmetric distortion in inorganic frameworks, amplifying overall film chirality.

Yuan et al. investigated solvent‐engineered crystallization dynamics in [(R)‐*β*‐MPA]_2_MAPb_2_I_7_ film fabricated by spin‐coating.^[^ ^119^ ^]^ Comparative studies of DMSO and DMF solvents revealed distinct growth mechanisms (Figure 7g). DMSO's high boiling point and strong coordination with MA⁺/PbI_x_^−^ intermediates promoted chiral cation adsorption on PbI_6_^−^ octahedra's (001) planes, replacing coordinated DMSO molecules and inducing in‐plane CHP growth. DMSO's polarity further facilitated hydrogen bonding with chiral ligands, stabilizing high‐dimensional phases. In contrast, DMF's rapid crystallization kinetics produced randomly oriented crystallites. Space‐charge‐limited‐current measurements confirmed superior carrier mobility in DMSO‐processed films compared to DMF counterparts (Figure 7h), highlighting the critical role of solvent‐mediated crystallization control in optimizing charge transport for high‐performance CHP optoelectronics. These studies unveil that tailored spin‐coating integrating antisolvent engineering and solvent selection can simultaneously enhance chiral activity and electronic properties in polycrystalline CHP films, offering a versatile platform for advanced photonic and electronic devices.

The design of micro/nanowire (MW/NW) architectures has been a promising strategy in CHPs engineering, leveraging morphology‐dependent advantages to enhance optoelectronic performance. The high surface‐to‐volume ratio of MWs/NWs improves light absorption by reducing reflection losses while providing direct pathways for carrier transport, thereby minimizing recombination and boosting photoconversion efficiency. Additionally, the exposed edges of these 1D structures facilitate exciton dissociation into free carriers, promoting efficient photocarrier generation and conduction. Zhang et al. attested to the potential of this approach by fabricating single‐crystal RP perovskite nanowires via capillary‐bridge assembly (CBA).^[^ ^120^ ^]^ Unlike polycrystalline films plagued by grain boundary defects, these NWs exhibited superior carrier mobility within their well‐ordered layered structures. Furthermore, periodic NW arrays can function as photonic crystals, manipulating light propagation to enhance absorption or emission at targeted wavelengths. For CHPs, the anisotropic optical/electrical properties intrinsic to NWs, such as linear polarization sensitivity, synergize with their inherent chirality‐driven CPL response, enabling integrated Stokes vector detection in a single device.

**Figure 8:**
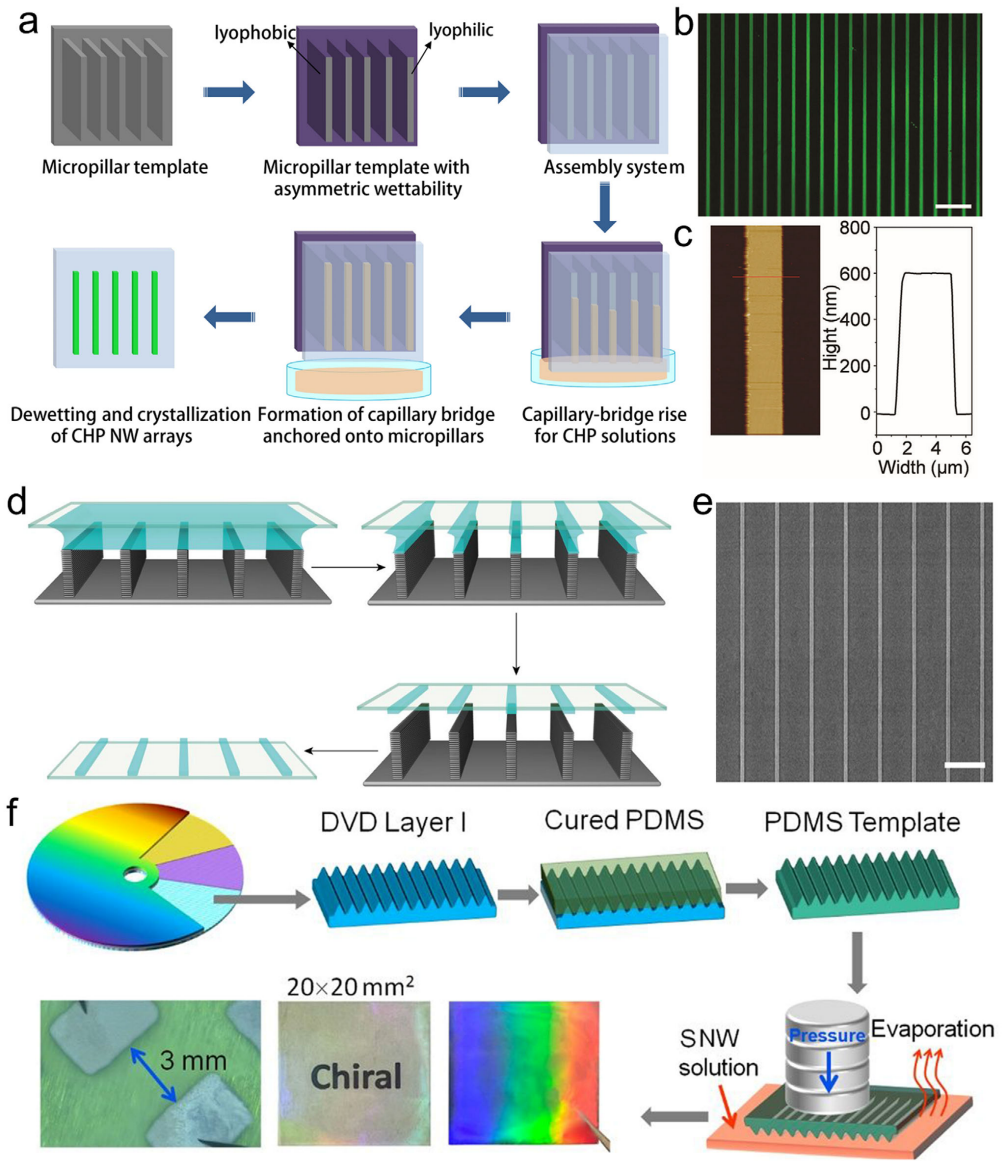
CBA and imprinting methods for CHP‐based devices. a) Schematic diagram of the fabrication of CHP microwires by the CBA method. b) Fluorescence microscopy image of microwire array. c) AFM of the single microwire. Reproduced with permission.^[^ ^121^ ^]^ Copyright 2021, Wiley‐VCH. d) CBA method for nanowire array. e) SEM of fabricated CHP NWs. Reproduced with permission.^[^ ^122^ ^]^ Copyright 2021, American Chemical Society. f) Imprinting process. Reproduced with permission.^[^ ^124^ ^]^ Copyright 2025, American Chemical Society.

In terms of the CBA approach, Zhao et al. synthesized (R/S‐MBA)_2_PbI_4_ NWs with high crystallinity and ordered crystallographic alignment, and the assembly system is sketched in **Figure** **8**a in detail.^[^ ^121^ ^]^ The micropillar templates were selectively functionalized by heptadecafluorodecyltrimethoxysilane (FAS) to create lyophobic sidewalls and lyophilic bottoms. The assembly system containing the template and flat substrate was perpendicularly placed on the CHP precursor. Subsequently, the solution rises into the gaps between the micropillar tops and the substrate under the combined action of capillary forces and Laplace pressure, forming discrete capillary bridges anchored at the micropillar surface. Single‐crystal NW arrays were defined on the substrate after the dewetting process. The uniform and bright green fluorescence of the array (Figure 8b) indicates that homogenous and high‐crystallinity CHP NWs are obtained. For clear observation of the NWs profile, AFM measurement was implemented, and the NWs exhibit a uniform height of 600 nm (Figure 8c). This approach leverages capillary force‐mediated precursor confinement and crystallization kinetics modulation, improving scalable fabrication of CHP NWs arrays with tunable optoelectronic properties.

Wu's group advanced the CBA strategy to achieve sub‐500 nm feature sizes.^[^ ^122^ ^]^ By engineering FAS‐modified templates with hydrophilic tops and hydrophobic sidewalls (Figure 8d), they confined precursor films that split into nanoscale capillary bridges. In detail, a liquid thin film is confined between the micropillar array and the substrate due to the contrasting wettability of the micropillar surfaces. When the solvent was evaporated, the continuous liquid film underwent capillary‐driven splitting, forming discrete capillary bridges anchored at the micropillar. Upon complete solvent removal, these bridges template the growth of parallelly aligned single‐crystalline 2D CHPs NW arrays. The SEM of the NWs array is shown in Figure 8e, the synthesized NWs show straight boundaries and a flat surface with a width of 480 nm. Through template‐directed programmable assembly, CHP NW arrays with tailored morphological dimensions (e.g., diameter, length) and areal density can be precisely engineered by adjusting the width and interpillar spacing of the micropillar template.

Complementing CBA, imprinting offers a low‐cost alternative for patterning nanostructured perovskite material. Imprinting utilizes the PDMS that duplicates the structure of the master template to obtain a patterned structure. Su et al. developed a PDMS‐based imprinting technique where precursor‐filled PDMS channels are aligned on substrates and annealed to form interfaces.^[^ ^123^ ^]^ In the construction, the solution was dropped into the channel of the PDMS, and the PDMS was aligned on the target, followed by an annealing process to obtain perovskite MW. Bao et al. utilized the DVD as a master template and copied the grating pattern by PDMS (Figure 8f).^[^ ^124^ ^]^ For fabricating moire‐structured NWs, the CHP solution was dropped onto the substrate, followed by an imprinting with high pressure. After the annealing process, the template was removed, and the NW array was prepared. Compared to CBA with nanoscale precision, the resolution of the imprinting method is subject to the master and is usually limited to micrometer‐scale resolution. This convenient fabrication facilitates interface‐optimized heterojunctions with other materials (such as metal oxides, and organic semiconductors), enhancing charge separation efficiency (e.g., CHP/ETL interface).

Both CBA and imprinting circumvent the complexity and cost of traditional lithography (e.g., photolithography, electron beam lithography), offering scalable routes for industrial applications. For CHPs, these methods open the door for defect‐minimized arrays with controlled linewidths, spacing, and orientation, which are keys for reducing carrier recombination. The rod‐like NW morphology additionally mitigates mechanical stress during bending, making CHP arrays compatible with flexible substrates (PET, PI) for wearable electronics and foldable displays. When combined with chirality‐driven functionalities, such architectures open avenues for high‐density, miniaturized devices like polarization‐sensitive PDs and mechanically robust chiroptical systems.

## 4 Novel Functions of CHPs

CHPs have been a cornerstone material for next‐generation chiral‐optoelectronic and spintronic technologies, capitalizing on their unique helical crystallographic frameworks, CISS, and intrinsic NLO properties. The synergy of structural chirality, SOC‐enhanced spin splitting, and spin‐selective scattering enables efficient spin‐polarized current generation, making CHPs promising for disruptive applications across diverse cutting‐edge domains, including CPL‐resolved PD, PAS, circularly polarized luminescence, spin‐polarized charge transport, circularly polarized SHG, and nonvolatile memory. The interplay of chirality‐mediated optical, electrical, magnetic, and spin degrees of freedom inspires unprecedented device functionalities characterized by ultracompact integration, ultralow power consumption, and dynamic reconfigurability. By transcending the performance limitations of conventional optoelectronic materials through multi‐physical field coupling, CHPs establish a versatile platform for next‐generation intelligent systems spanning quantum photonics, neuromorphic computing, and adaptive opto‐spintronic architectures.

### 4.1 CPL Detectors of CHPs

**Figure 9:**
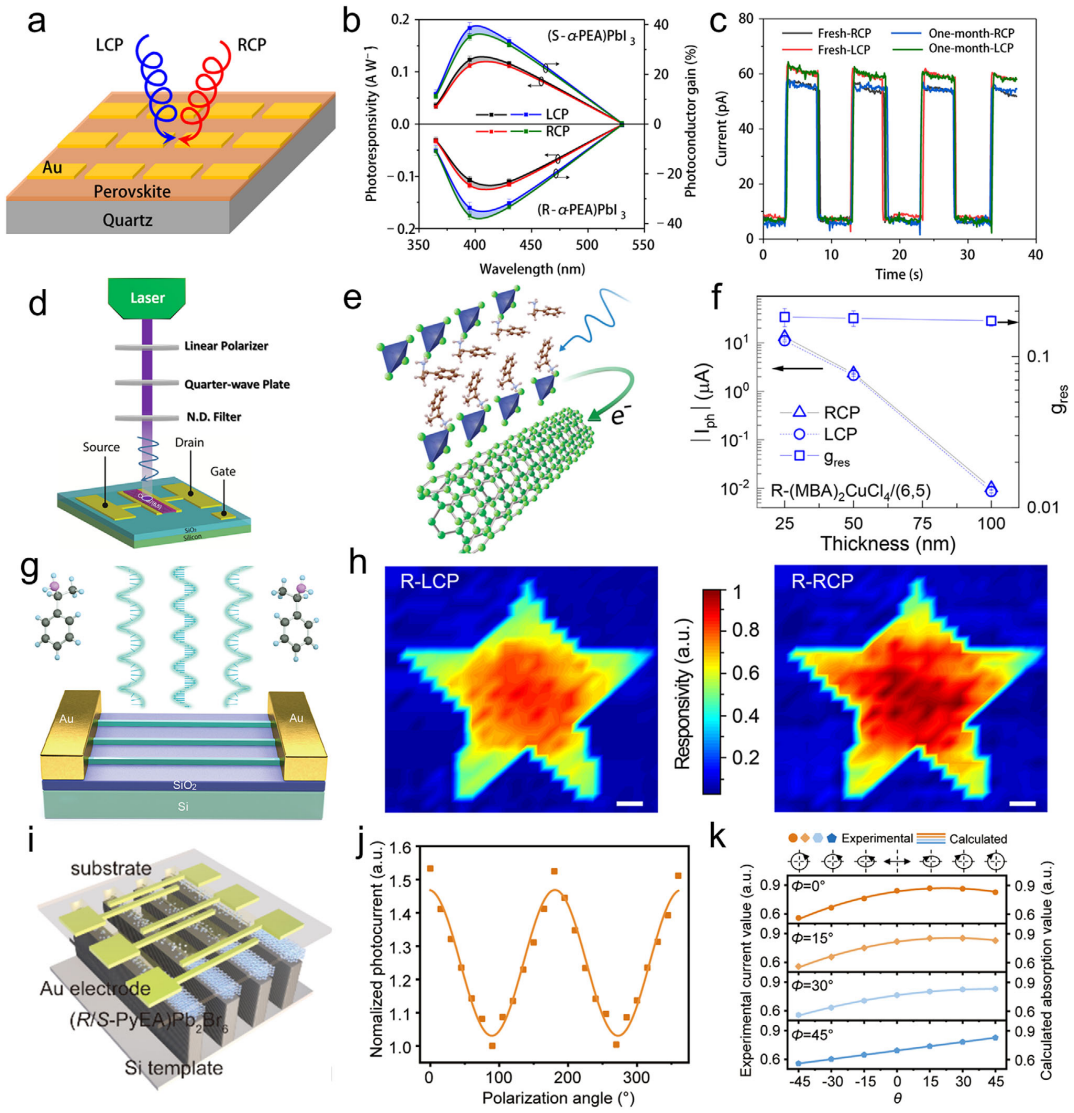
CPL‐resolved PDs based on CHPs. a) Schematic of (R‐and S‐α‐PEA)PbI_3_ film PD. b) Responsivity and photoconductor gain under different wavelengths. c) Stability test of the CHP‐based PD. Reproduced with permission.^[^ ^42^ ^]^ Copyright 2019, Nature Publishing Group. d) Schematic diagram of CHP/SWNT heterostructure CPL PD. e) Carrier dynamics inside the heterostructure. f) Response of the device with different CHP thicknesses. Reproduced with permission.^[^ ^125^ ^]^ Copyright 2021, American Chemical Society. g) CHP NW‐based PD. h) Imaging by CHP NW under the illumination of LCP (left) and RCP (right). Reproduced with permission.^[^ ^122^ ^]^ Copyright 2021, American Chemical Society. i) Schematic illustration of Au/CHP/Au PD structure. j) Polarization‐dependent photocurrent of the R‐type device under illumination (365 nm, 69 mW cm^−2^). k) Measured and calculated Stokes vectors. Reproduced with permission.^[^ ^47^ ^]^ Copyright 2024, American Chemical Society.

The helical structure of CHPs breaks spatial symmetry, endowing them with CD values far exceeding those of traditional chiral organic materials. This strong asymmetry enables CHPs to exhibit distinct absorption of left‐handed and right‐handed circularly polarized light (LCP/RCP). Figures of merit for evaluating optical detection performance include responsivity(*R*), detectivity(*D* ^*^), and response time, among others. *R* reflects the capability of the detector in converting optical signals into electrical signals, and a large *R* value indicates a high sensitivity toward weak light. *R* is calculated by *R* = *ΔI*/(*P* × *S*), where *ΔI* is the difference between photocurrent and dark current (i.e., *I*_light_ – *I*_dark_), *P* is power of the incident light, and *S* is the effective area. *D* ^*^ is utilized to characterize the performance of detectors with different sizes and bandwidths, which represents the response resolution of the PDs. *D* ^*^ is expressed as D* = (*S* ×  Δ*f*)^1/2^/*NEP*, where *S* is the exposed area, *Δf* is operating bandwidth, and *NEP* (*I*_noise_/*R, I*_noise_ is the noise current) is the noise equivalent power. Response time (µ) is usually characterized by subjecting the detector to a pulsed light source and recording the time it takes for the output signal to rise from a low level to its steady state (rise time) or decay back to the baseline (fall time). It shows the response speed of the detector toward variations of external signals. Tang et al. capitalized on this property by developing a planar (R/S‐α‐PEA)PbI₃‐based PD with exceptional responsivity of 797 mA W⁻¹ and detectivity of 7.1 × 10¹¹ Jones (**Figure** **9**a).^[^ ^42^ ^]^ The α‐PEA ligand, featuring conjugated π bonds, enhances Coulomb interactions between chiral cations and [PbI_6_]^4−^ octahedra, amplifying CPL discrimination. Wavelength‐dependent responsivity and photoconductor gain of the PD toward various wavelengths (365, 395, 430, and 530 nm) are summarized in Figure 9b. Responsivity peak (0.12 A W^−1^) could be observed at 395 nm, indicating the best distinguishing ability for 395 nm LCP and RCP. However, the difference in other wavelengths is not obvious, and the difference disappears at 530 nm, which is identical to the absorption and CD spectra. In addition to excellent CPL response behavior, the proposed CHP‐based PD also exhibits superior stability. A comparison between the fresh device and the old device (in ambient after one month) is performed, and the consistent response toward 395 nm CPL (Figure 9c) indicates that the device shows no degradation. This work proposes a CPL PD with remarkable optoelectronic performance and strong stability, establishing a pathway for miniaturized polarization‐resolved imaging systems with robust operational longevity.

Despite their superior chiroptical activity, CHPs suffer from limited conductivity due to insulating chiral organic layers. Hybrid architectures combining CHPs with high‐mobility materials address this limitation by optimizing charge transfer. Blackburn et al. ascertained this strategy through an (R‐/S‐MBA)_2_CuCl_4_/single‐wall carbon nanotube (SWNT) heterostructure in a CPL‐sensitive FET.^[^ ^125^ ^]^ Figure 9d shows the setup for the CPL measurement. The light with a random direction passes through a linear polarizer and a quarter‐waveplate to generate CPL, and the neutral density filter controls the output intensity. This heterostructure leverages spin‐selective interfacial charge transfer (Figure 9e), which combines the significant chiroptical response of the CHP absorb layers with the excellent carrier transport properties of the SWNT channel. Circularly polarized photons are initially absorbed by the CHP, followed by an ultrafast electron transfer to the SWNT layer, achieving record polarization‐dependent responsivity of 452A/W. Thickness optimization studies (Figure 9f) revealed a three‐order‐of‐magnitude photocurrent enhancement in 25 nm CHP films versus 100 nm counterparts, attributed to reduced carrier recombination in thinner layers. These optimized performance metrics based on CHP/SWNT hybrid semiconductor architectures rival those of state‐of‐the‐art CPL detectors, highlighting the potential of an interfacial engineering strategy for developing advanced chiral optoelectronics. The enhanced functionality stems from the spin‐selective charge transport mechanism achieved through precisely engineered nanoscale heterojunction interfaces, which establishes a new paradigm for manipulating chiral‐induced spin polarization in tailored material systems. The widespread adoption of CHPs has been hindered by the toxicity of lead in their composition. Addressing this challenge, Luo et al. proposed lead‐free chiral halide double perovskite [(R)‐β‐MPA]_4_AgBiI_8_, which combines phase stability with exceptional photophysical properties, expanding its application in self‐powered CPL photodetection.^[^ ^126^ ^]^ The chiral polar crystal structure of this material facilitates a bulk photovoltaic effect, generating a built‐in electric field that drives the spontaneous separation of photogenerated carriers. This mechanism eliminates the need for external bias, achieving zero‐bias CPL detection with a high anisotropy factor (≈0.3) and ultra‐low power consumption. The device demonstrated reproducible optical switching under illumination and retained robust polarization discrimination over extended periods, reliably distinguishing between LCP, RCP, and unpolarized light. By integrating lead‐free design with efficient chirality‐driven carrier dynamics, this work establishes a sustainable pathway for eco‐friendly and energy‐efficient polarization‐sensitive optoelectronics.

Full‐Stokes polarimetry, requiring simultaneous detection of intensity (S₀), linear polarization (S₁/S₂), and circular polarization (S₃), is critical for applications ranging from quantum communication to biomedical imaging. Conventional systems rely on bulky optical components (e.g., waveplates, polarizers), suffering from inefficiency and integration challenges. CHPs offer an alternative by combining intrinsic CD with engineered structural anisotropy to resolve all Stokes parameters in compact architectures. Zhao et al. pioneered this approach using an aligned (S‐α‐PEA)_2_PbI_4_ NW array (Figure 9g), achieving dual polarization sensitivity via crystallographic anisotropy and chiral interlayer interactions.^[^ ^122^ ^]^ High crystallinity and pure crystal orientation allow fast in‐plane carrier transport along the NW direction, generating a high responsivity (47.1 A W^−1^) and detectivity (1.24 × 10^13^ Jones). Relying on the chiral cations intercalated between halide perovskite layers, a high g_CD_ of 0.15 is achieved. The strictly aligned NWs are responsive to CPL with obvious response differences. As shown in Figure 9h, the five‐point star region that is exposed to illumination shows shape edges compared to the unexposed area, and the center shows obvious variation toward LCP and RCP, demonstrating excellent CPL‐resolved ability of the CHP NWs device. In terms of the anisotropic dielectric role, the CBA NWs display a high linear polarization ratio of 1.6. The sensing of the Stokes parameters was further verified on the CHP NWs array, providing a new platform for constructing compact polarization detectors.

Bai et al. extended this strategy to a large‐scale (R/S‐PyEA)Pb_2_Br_6_ NW array and achieved 256‐pixel CPL imaging and full‐Stokes detection.^[^ ^47^ ^]^ Figure 9i shows the Schematic of the Au/CHP NWs/Au array, in which the CHP NW is deposited on the silicon template. The CHP NWs possess a small exciton binding energy of 57.3 meV, ultra‐high responsivity of 86.7 A/W, remarkable detectivity of 4.84 × 10^13^ Jones, and high g_CD_ of 0.42. In terms of the anisotropic structure‐induced absorption difference, the CHP NWs yield different photocurrent responses (Figure 9j). In this measurement, the zero degree is defined as the direction that is parallel to the growth of NWs. With the linear polarized light rotating from 0° to 180°, a sinusoidal function‐like variation could be observed, exhibiting typical angle‐dependent behavior. The linear dichroic ratio is calculated by *I*_max_/*I*_min_, and a high value of 1.52 is obtained, indicating superior distinguishability toward linear polarization. The discrimination features for both linear polarization and circular polarization underlie the basis for the full‐Stokes polarimeter. The measured Stokes parameters (S_0_–S_3_) are plotted in Figure 9k, which are consistent with the theoretically calculated result, confirming the achievement of all‐in‐one polarization PD.

Yuan et al. enhanced the structural chirality of CHP by mixing halides, significantly boosting their chiroptical activity, which promotes the development of high‐performance and self‐powered full‐Stokes polarimetry devices.^[^ ^127^ ^]^ High‐throughput first‐principles calculations reveal that the magnetic transition dipole moment (∣mab∣) of mixed halide perovskites is significantly enhanced. This enhancement results in g_CD_ increasing from 0.0016 in pure halides to 0.025 in mixed halides, marking a 16‐fold amplification. The carrier mobility (2.64 × 10⁻^2^ cm^2^ V⁻¹ s⁻¹) and detectivity (1.2 × 10¹^2^ Jones) of mixed halide perovskites (such as (S‐MBA)₄Bi₂I₅Br₅) are significantly superior to those of pure halide systems, with a low dark current drift (2.52 pA µm⁻¹ s⁻¹ V⁻¹), indicating their outstanding stability. Moreover, chiral polarity leads to an intrinsic electric field (open‐circuit voltage of 0.50 V), meaning operation without the need for external bias. The error in detecting the Stokes parameters of mixed halide devices is ΔS₁ = 3.8%, ΔS₂ = 4.0%, ΔS₃ = 5.0%, surpassing existing semiconductor‐based polarization detectors. A 20 × 20 pixel full‐Stokes imaging has been achieved, accurately matching the input signal with a linear degree of polarization (DOP) of 0.50 and a circular degree of polarization of 0.86. Through a halide mixing strategy, this study successfully enhanced the structural chirality distortion of perovskites, achieving highly sensitive self‐powered full‐Stokes polarization detection and imaging. This advancement promotes the widespread application of CHPs in the field of polarization detection.

The inherent chiral architecture of halide perovskites endows these materials with unique capabilities for discriminating CPL, primarily through pronounced CD. Combined with their exceptional broadband absorption coefficients and high charge carrier mobilities, CHPs support direct CPL‐to‐electric signal conversion, achieving superior sensitivity and sub‐millisecond response speeds compared to conventional polarization detection methodologies. CHP‐based PDs exhibit tremendous advantages in miniaturization, mechanical flexibility, fabrication cost, and energy efficiency. Unlike traditional CPL detection systems that require cascaded optical components (e.g., quarter‐wave plates, linear polarizers) with complex alignment, CHP devices leverage intrinsic chiral‐material properties to decode polarization states in compact architectures, encouraging seamless integration into flexible substrates or photonic integrated circuits. Furthermore, compositional engineering via halide alloying or organic cation substitution allows precise bandgap tuning across the visible to near‐infrared spectrum (400–1000 nm), bypassing the wavelength limitations of conventional optics dictated by fixed waveplate materials. Solution‐processable fabrication techniques (spin‐coating, inkjet printing) further distinguish CHPs from traditional systems reliant on expensive high‐precision machining (e.g., quart‐waveplates), offering a cost‐effective route for scalable production. The bulk photovoltaic effect inherent to polar CHP crystals realizes self‐powered CPL detection without external bias, reducing operational energy demands by orders of magnitude compared to voltage‐dependent detectors.

Despite these merits, critical challenges hinder practical deployment. CHPs remain vulnerable to humidity‐induced degradation, necessitating advanced encapsulation strategies to ensure long‐term operational stability. Environmental concerns associated with lead toxicity in prototypical systems persist, while lead‐free alternatives (e.g., Ag/Bi‐based CHPs) currently underperform in terms of both chiroptical activity and charge transport metrics. Future endeavors could concentrate on the following interconnected priorities. Chiral crystalline frameworks should be improved through defect passivation and hydrophobic coatings. Meanwhile, lead‐free CHPs with competitive *g*_CD_ and carrier mobilities should be developed. The heterojunction interfaces and device architectures for complementary metal‐oxide semiconductor (CMOS) compatible integration deserve further optimization. Addressing these challenges will unlock the full potential of CHPs in next‐generation optoelectronic applications spanning quantum communication, wearable biosensors, and autonomous machine vision systems.

For intuitional study of emerging CHPs in sensing fields, a list of CHPs‐based PDs, including the chemical formula, device structure, responsivity (R), response wavelength (RW), and chirality degree indicated by CD value, is summarized in **Table** **1**.

**Table 1:** Chiral halide perovskite‐based photodetectors.

Formula	Structure	R	RW (nm)	CD (mdeg)	Refs.
(R‐PEA)PbI_3_	Au/CHP/Au	797 mA W^−1^	395	≈200	[42]
(R‐NEA)PbI_3_	Ag/BCP/CHP/SnO_2_/TCO	0.28 A W^−1^	395	3000	[81]
(R‐MPA)_2_MAPb_2_I_7_	Au/CHP/Au/PET	3.8 A W^−1^	532	40	[119]
(S‐MBA)_2_CuCl_4_	Au/CHP/SWNT/Au	452 A W^−1^	405	<100	[125]
(R‐NEA)_2_PbI_4_	Au/CHP/Au	15.7 A W^−1^	500	<150	[128]
(S‐NEA)PbI_3_	Ag/PCBM/CHP/HTL/ITO	1 mA W^−1^	400	≈2000	[129]
(R‐BPEA)_2_PbI_4_	Au/CHP/MAPbI_3_/Au	–	500	20	[130]
(R‐MBA_0.5_nBA_0.5_)PbI_4_	Al/BCP/PCBM/CHP/PEDOT: PSS/ITO	142 mA W^−1^	503	≈150	[131]
(R‐NEA)PbI_3_	Au/CHP/Au	35 mA W^−1^	405	40	[132]
[S−3APr]PbI_4_	Au/CHP/Au	1.97 A W^−1^	500	<10	[133]
(R‐PEA)_2_PbI_4_	Au/CHP/MAPbI_3_/Au	4.26 mA W^−1^	505	60	[134]
(S‐ MPA)EAPbBr_4_	Ag/CHP/Ag	‐	405	<50	[135]
(R‐ClPEA)_0.6_HDA_0.7_PbI_4_	Au/MoO_3_/CHP/ITO	33 mA W^−1^	483	<40	[136]
(S‐PEA)_2_FAPb_2_I_7_	Au/CHP/Au	0.04 mA W^−1^	785	<20	[137]
(S‐PyEA)Pb_2_Br_6_	Au/CHP/Au	86.7 A W^−1^	365	<25	[138]
(R‐MBA)_2_MAPb_2_I_7_	Ag/PCBM/CHP/NiO_x_/ITO	0.82 A W^−1^	505	12	[139]
R‐(BrBA)_2_PbBr_4_	Ag/CHP/Ag	–	405	50	[140]
(S‐MBA)_4_AgBiI_8_	Au/CHP/Au	52 mA W^−1^	520	<25	[141]
(R‐MBA)_4_AgBiI_8_	Au/CHP/Au	–	450	<200	[142]

### 4.2 CPL‐Sensing PAS using CHPs

In PD devices, photogenerated electron‐hole pairs undergo rapid separation at the perovskite layer or heterojunction interface, followed by efficient collection at electrodes to generate transient photocurrent. The system exhibits reversible behavior, returning to its baseline state immediately upon illumination removal, with no inherent memory functionality. In contrast, PAS utilizes defect‐mediated carrier dynamics within perovskites, and photoinduced charges become trapped at intrinsic defect sites, inducing persistent modifications in device conductivity. These conductivity states demonstrate nonvolatile retention over extended durations, effectively emulating the memory characteristics of biological synapses. Crucially, synaptic weight modulation can be dynamically programmed through temporally coded light pulse sequences, enabling sophisticated spatiotemporal information processing capabilities. PAS, leveraging the high bandwidth, ultralow crosstalk, and multidimensional tunability of light‐matter interactions, are driving a paradigm shift in neuromorphic engineering, transitioning from conventional electrical modulation to advanced photonic modulation frameworks.^[^ ^143^, ^144^, ^145^ ^]^ Polarization encoding further enriches optical information capacity by incorporating higher‐order parameters such as ellipticity, azimuthal angle, and helicity.^[^ ^146^, ^147^, ^148^ ^]^ Among these, CPL‐resolved PAS devices with integrated in‐sensor computing capabilities represent a breakthrough for real‐time vision systems in terms of parallel processing of complex optical stimuli.^[^ ^149^, ^150^ ^]^ CHPs uniquely synergize chirality‐governed spin‐selective phenomena with exceptional optoelectronic properties (e.g., tunable exciton dynamics, anisotropic carrier transport), offering a biomimetic platform for multi‐dimensional sensing and energy‐efficient photonic synapses. These devices exploit the interplay between chiral‐induced asymmetric carrier injection and polarization‐dependent exciton dissociation to emulate synaptic plasticity under ultralow power consumption. By encoding polarization states into spatiotemporal synaptic weights, CHP‐based PAS systems open new pathways for adaptive neuromorphic computing, edge‐enabled machine vision, and photonic neural networks with inherent parallelism and wavelength‐division multiplexing capabilities.

**Figure 10:**
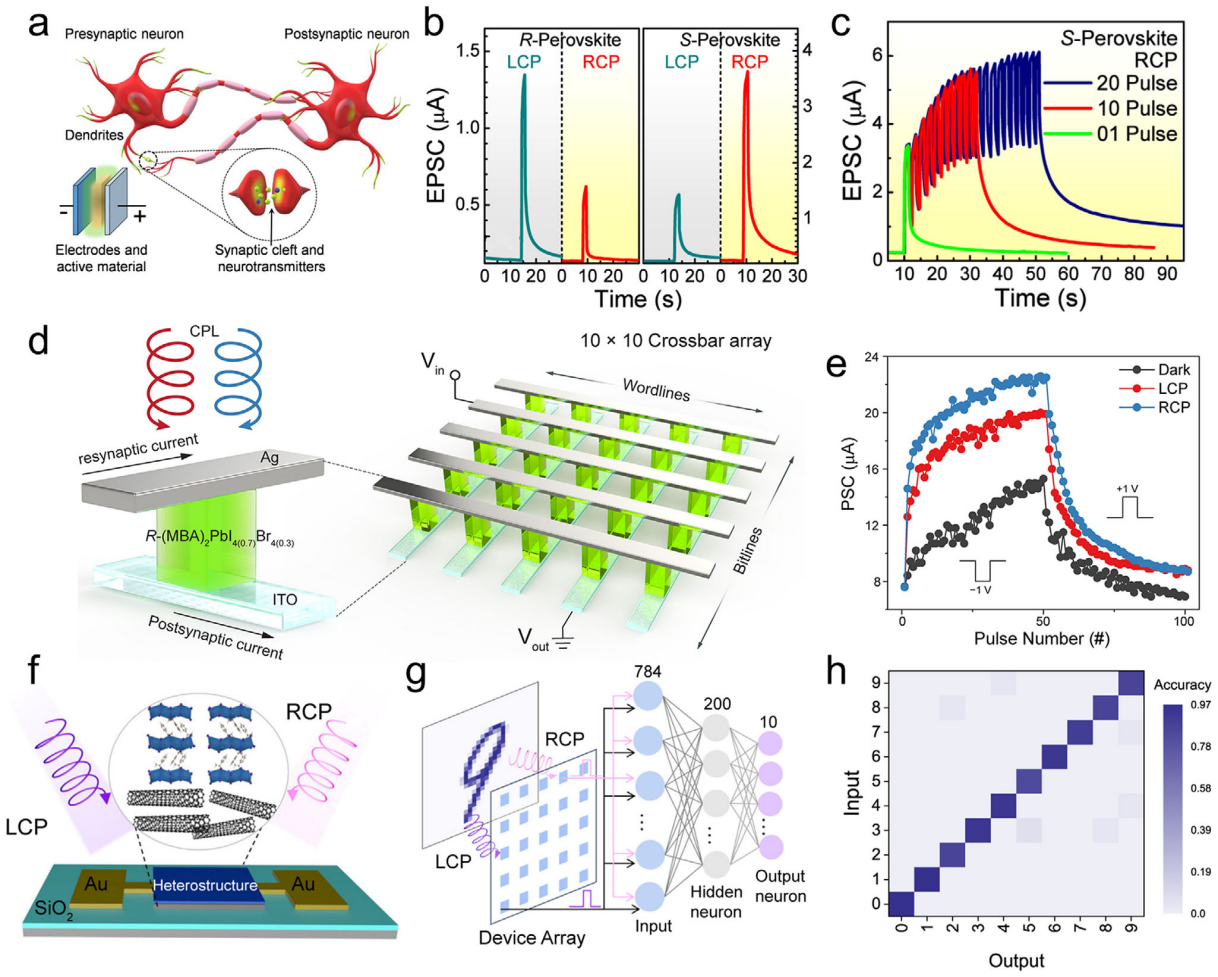
CPL‐resolved PAS devices. a) Schematic of the biological and artificial synapse. b) EPSC of R‐type and S‐type PAS in response to LCP and RCP stimulation. c) EPSC response under multiple light pulses. Reproduced with permission.^[^ ^151^ ^]^ Copyright 2024, American Chemical Society. d). PAS array and corresponding light modulation method. e). LTP and LTD under different stimulation conditions. Reproduced with permission.^[^ ^152^ ^]^ Copyright 2024, American Chemical Society. f) SWNT/CHP heterostructure PAS device g) Simulated SNN based on the PAS device. h) Recognition results output by the trained SNN. Reproduced with permission.^[^ ^46^ ^]^ Copyright 2023, Nature Publishing Group.

Recent advances in neuromorphic engineering have yielded CHP‐based PAS devices that emulate synaptic plasticity through polarization‐encoded optical stimuli. Pal et al. developed a bio‐inspired ITO/CHP/PMMA/Al structured PAS (**Figure** **10**a), where the CHP layer functions as a polarization‐sensitive synaptic cleft.^[^ ^151^ ^]^ Excitatory postsynaptic current (EPSC) triggered by a single LCP/RCP spike was successfully imitated by the R‐type and S‐type devices (Figure 10b), respectively. For an R‐type device, a higher EPSC peak is observed under the stimulation of the LCP beam, and the response gap indicates that the PAS device is capable of discriminating the circular polarization states. The S‐type PAS exhibits similar CPL distinguishing ability but an opposite response preference. Short‐term plasticity (STP) can be converted to long‐term plasticity (LTP) by increasing the stimulus CPL spikes, and Figure 10c sketches the transition by performing a sequence of spikes. The EPSC response of the PAS device depends on both the current input stimuli and the historical state and generally exhibits an ascending trend with the increase of CPL spikes.^[^ ^153^, ^154^ ^]^ To elucidate the synaptic behavior, a charge trapping‐detrapping mechanism was proposed and verified by Kelvin probe force microscopy (KPFM). In detail, the photoinduced holes can escape the CHP layer and leave the electrons in the conduction band, which decreases the Schottky barrier at the ITO/CHP interface, leading to the potentiation behavior (conductivity enhancement). Without illumination, the quantum wells in the CHP trapping the carriers delay the electron‐hole recombination and induce a slow decay of the EPSC behavior. Hwang and co‐workers designed the PAS with a FET structure of Au/pentacene/PMMA/CHP/SiO_2_/Si.^[^ ^155^ ^]^ In the intermediate functional layer, the CHP generates circular polarization‐dependent photocurrent, and the pentacene, as carrier extraction layer, implements the charge separation for memory properties, showcasing CPL‐dependent synaptic characteristics. Owing to the heterostructure design, the PAS device achieves a high g_CD_ of 0.3 and responsivity of 130 mA W^−1^. Further, pattern recognition was verified on a constructed 3 × 4 PAS array by tuning the handedness of CPL and stimulation interval, broadening applications of CPL‐resolved PAS in neuromorphic computing. Park et al. fabricated a cross‐point PAS array with a vertical structure of Ag/CHP/ITO (Figure 10d).^[^ ^152^ ^]^ This array could directly execute the matrix‐vector multiplication (MVM) in terms of Ohm's Law and Kirchhoff's Law, which accelerates the calculation and reduces the power consumption.^[^ ^98^, ^156^, ^157^ ^]^ In the crossbar array, each cross‐point corresponds to a memory cell, with its conductance value *G_ij_* programmable to the matrix element value. An input voltage vector *V_j_* is applied to the word lines, while the output current is read from the bit lines according to the equation *I_i_* =  *G_ij_V_j_*, directly performing MVM in the analog domain.^[^ ^158^, ^159^, ^160^ ^]^ The MVM operation features one‐shot computation of an 𝑁 × 𝑁 matrix multiplication, with the array size determining the value of 𝑁. By leveraging the inherent parallelism and analog computation capabilities, this method extremely reduces energy consumption compared to traditional computing systems, where analog‐to‐digital conversion (ADC) operations typically account for over 60% of the total energy expenditure.^[^ ^161^, ^162^ ^]^ Multilevel programmable conductance states of PAS inside the crossbar architecture serve an important role in the accurate in‐memory computing hardware platform.^[^ ^163^, ^164^ ^]^ As shown in Figure 10e, the PAS exhibits multiple long‐term potentiation and long‐term depression states of 50, and the circular polarization‐resolved capability further enhances the tunability of those states. Based on the PAS array, the artificial neural network (ANN) was simulated and achieved an accuracy of 82% under the modulation of LCP. This work pioneers a hardware‐level in‐memory computing paradigm by convergently engineering polarization modulation and nonvolatile memory elements, providing a blueprint for achieving PAS with unprecedented energy efficiency and ultrafast computation speed.

Li et al. proposed a CPL‐resolved ultraviolet PAS based on CHP/SWNT heterostructure (Figure 10f), and systematically exploited the underlying mechanism and potential application.^[^ ^46^ ^]^ The heterostructures exhibit excellent polarization‐dependent photoresponsivity, and are capable of distinguishing LCP and RCP beams. With the aid of the femtosecond pump‐probe spectroscopy, a spike‐timing dependent transient absorption (TA) observation method is designed to study the carrier dynamics inside the heterostructure. The detected ultrafast carrier dynamics, combined with energy band analysis, explicates the hole transfer process existing in the heterostructure, which contributes to a bioinspired PAS. A series of polarization‐dependent synaptic behaviors are successfully imitated by the PAS device, and the developed PAS device array successfully achieves perception, learning, and recognition. To evaluate the performance of the PAS in artificial neural networks, pattern recognition, which is trained on spike neural networks (SNNs) with PAS modeling as spiking neurons, is simulated. In the SNNs, the input data set is converted to spikes and passed to the neural network. The spiking neuron collects the weighted sum of input spikes and forms the membrane potential of the neuron. Under sufficient excitation, the membrane potential will reach a threshold (θ) and release a spike to its next connections.^[^ ^165^, ^166^ ^]^ Typically, the membrane potential has a decay over time owing to the short burst and uncertain emission time of input spikes, and PAS possesses analogous temporal dynamics, allowing it to construct the bioinspired SNNs. Figure 10g shows the SNNs architecture consisting of 784 inputs, 200 hidden, and 10 output neurons. In the evaluation, the Modified National Institute of Standards and Technology (MNIST) database is selected as the training and test target. In detail, the recognition accuracy of SNNs model is evaluated by 60 000 training and 10 000 test images with a batch size of 200 at each epoch. Eventually, the PAS‐based SNNs show a relatively high recognition accuracy of 0.93 after 200 epochs. Further, the recognition for all datasets is summarized as shown in Figure 10h, and it can be seen that every kind of dataset can be recognized with high accuracy, demonstrating that the PAS‐based SNNs have excellent classification ability after the training. The constructed PAS with CPL‐resolved features improves the adaptability of photonic synapses and provides a new method for polarization‐based bioinspired computing paradigms. Polarization‐dependent PAS devices with high biological plasticity are advantageous in constructing advanced vision systems owing to their unique in‐sensor computing ability.^[^ ^167^, ^168^ ^]^ However, current polarization‐resolved PAS devices are restricted to monotonous recognition capability toward linearly polarized light or CPL. Liu et al. proposed a CHP NW‐based heterostructure to achieve linear and circular polarization‐sensitive all‐in‐one PAS devices.^[^ ^169^ ^]^ The designed PAS device exhibits high CD (400 mdeg) from chiral materials and intrinsic linear dichroism from nanowire morphology. TA spectroscopy confirmed prolonged carrier lifetimes due to interfacial hole transfer to MXene, engendering high responsivity (2.2 A W⁻¹) and multilevel synaptic plasticity. In application, the PAS device‐based reservoir computing is performed and exhibits good prediction in the chaotic forecasting task, achieving a normalized root mean square error of 0.023. This dual‐polarization sensitivity permits simultaneous extraction of Stokes parameters (S₁‐S₃) during optical preprocessing, laying the foundation for polarization‐multiplexed neuromorphic vision systems.

CHPs have emerged as a groundbreaking class of materials by integrating the intrinsic chiroptical properties of chiral systems with the exceptional optoelectronic performance of halide perovskites. This synergy has propelled CHPs into the spotlight for advanced applications in photonic energy conversion and high‐speed information processing. Among their innovative uses, circular polarization‐resolved PAS represents a frontier in neuromorphic device development. These devices exploit the polarization‐sensitive light‐matter interactions and structural tunability of CHPs to attain biomimetic detection and parallel processing of CPL signals, effectively emulating the synaptic plasticity and adaptive learning functions of biological neural networks. By leveraging the innate polarization selectivity of CHPs, circularly polarized synaptic devices can mimic sophisticated biological processes, such as the retina's perception of light directionality and polarization, thereby enhancing computational efficiency in tasks like image recognition. Their ability to process multidimensional optical signals, including intensity, wavelength, and polarization, offers unprecedented parallelism and energy efficiency, making them ideal for brain‐inspired computing architectures in real‐time decision‐making and complex pattern recognition. Unlike conventional optical synapses, CHP‐based PAS systems introduce light polarization as an additional control parameter, significantly expanding the design space for information encryption, reducing power demands, and improving resilience to optical noise. This multidimensional signal handling also positions CHP‐PAS devices as critical components in next‐generation optical neural networks, SNNs, and reservoir computing frameworks. Such systems hold immense promise for advancing artificial intelligence and bio‐inspired computing, potentially revolutionizing intelligent sensing, adaptive robotics, and ultra‐low‐power neuromorphic processors. This integration of chirality‐driven photonics with perovskite optoelectronics not only addresses current limitations in traditional computing architectures but also opens avenues for multifunctional, biohybrid systems that bridge materials science, neuroscience, and machine learning.

### 4.3 Circularly Polarized Emissions and LEDs of CHPs

Circularly polarized luminescence devices based on CHPs typically employ a multilayer design comprising a transparent conductive substrate (ITO), ETL (PCBM, TiO₂, and SnO₂), CHP luminescent layer, HTL (Spiro‐OMeTAD and PEDOT: PSS), and metal electrodes. The exceptional CPL performance originates from the intrinsic SOC within the CHP lattice. Here, symmetry breaking, induced by chiral organic ligands or distorted inorganic frameworks, generates a pronounced Rashba band splitting. This splitting produces spin‐polarized charge carriers during exciton recombination, while the CISS effect directly converts electron spin angular momentum into photon circular polarization. Strong photon–exciton coupling further amplifies the g_lum_, enabling devices to achieve external‐optics‐free, wavelength‐tunable, and high‐efficiency CPL emission.

This unique structure‐property interplay positions CHP‐based CPL devices as prominent light sources for next‐generation technologies. Their ability to intrinsically generate and modulate CPL, without relying on bulky optical components, makes them ideal for applications demanding compact, tunable, and high‐purity CPL, such as quantum information encryption and ultrahigh‐resolution 3D displays. By unifying chiral photonic control with perovskite optoelectronics, these systems open pathways for multifunctional, miniaturized photonic platforms that bridge quantum optics, spintronics, and bio‐inspired sensing.

**Figure 11:**
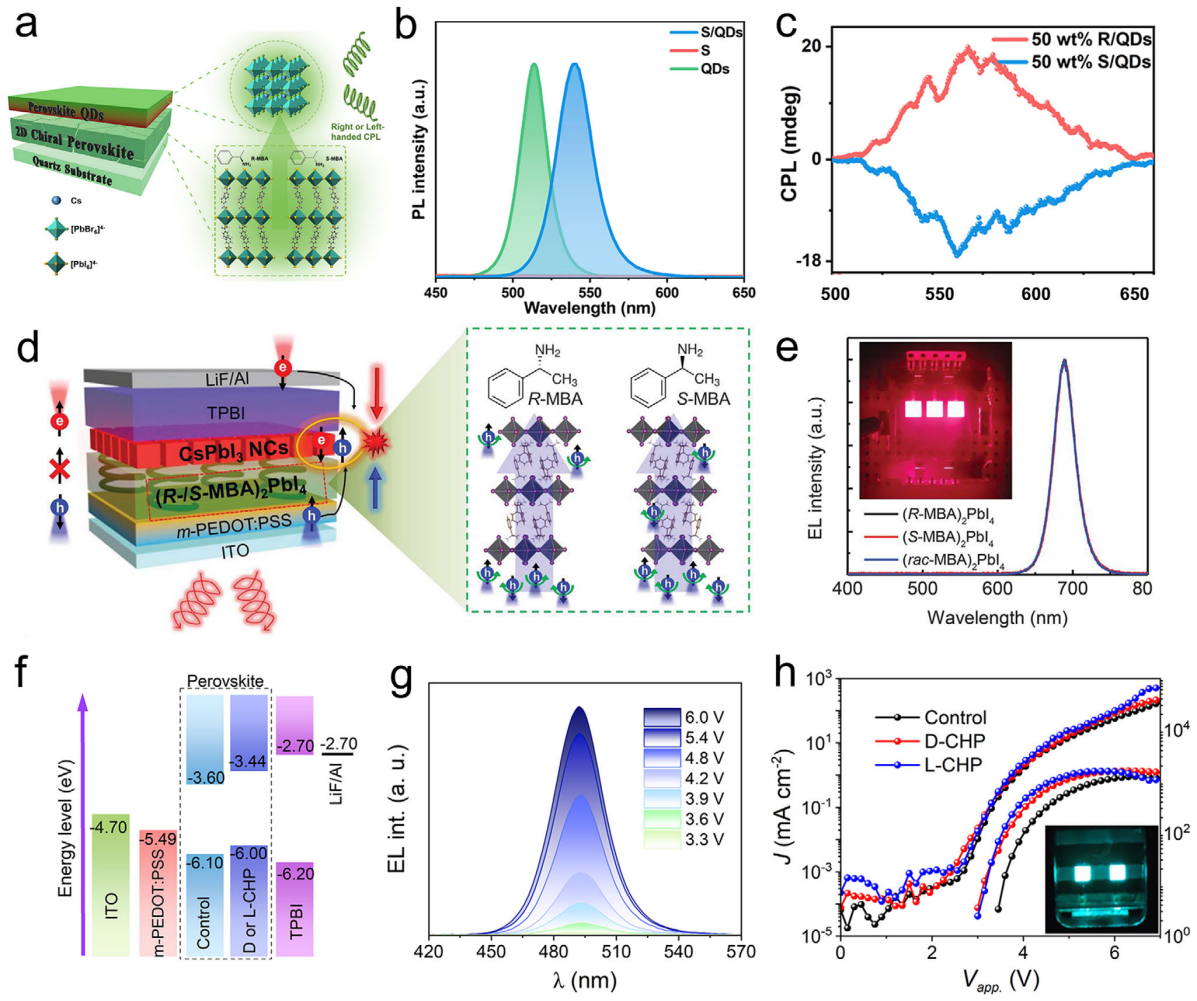
Circularly polarized PL and LEDs. a) Schematic for the structure of CsPbBr_3_ QDs and 2D CHP heterostructure. b). PL spectra of the heterostructure, CHP film, and CsPbBr_3_ QDs. c) Circularly polarized luminescence spectra of R‐type and S‐type heterostructure. Reproduced with permission.^[^ ^170^ ^]^ Copyright 2024, American Chemical Society. d) CP‐EL in CHP‐based device. e). EL spectra and spin‐LEDs. Reproduced with permission.^[^ ^45^ ^]^ Copyright 2021, AAAS. f) Energy band for the spin‐LEDs device. g) EL spectra under different bias voltages. h) Relationship between current density, voltage, and luminescence. Reproduced with permission.^[^ ^171^ ^]^ Copyright 2025, Wiley‐VCH.

Two‐dimensional CHPs exhibit strong CD signals, however, their optoelectronic utility is limited by severe non‐radiative recombination, which quenches PL and circularly polarized luminescence activity. Addressing this challenge, Wang et al. pioneered an innovative heterostructure design that synergizes 2D CHP with CsPbBr_3_ QDs, unlocking unprecedented CPL performance.^[^ ^170^ ^]^ The team synthesized 2D CHP by intercalating chiral organic molecules (R/S‐MBA) into a lead iodide perovskite lattice, inducing a chiral arrangement in the inorganic framework. Concurrently, cubic‐phase CsPbBr_3_ QDs (≈10 nm diameter) were prepared via solution processing. The heterostructure was fabricated by spin‐coating CsPbBr_3_ QDs (dissolved in n‐hexane) onto the 2D CHP film, forming a vertically stacked architecture (**Figure** **11**a). Halogen anion exchange at the interface (I^−^ with Br≈) transformed the QDs into mixed‐halide CsPbBr_3x_I_3(1−x)_, red‐shifting the PL emission (Figure 11b) from 515 nm (pristine QDs) to 540 nm (heterostructure). By modulating the CHP layer thickness or halide composition (e.g., R/S‐MBA)_2_PbBr_x_I_4(1−x)_), the team achieved precise control of emission wavelengths (550–647 nm) and g_lum_ up to 6 × 10^−3^. The spin polarization imbalance leads to the emission of CPL during the radiative recombination of the QDs layer, with the intensity positively correlated to the thickness of the CHP layer. Figure 11c shows the circularly polarized luminescence with the concentration of CHP precursor reaching 50 wt.%. TA spectroscopy revealed excitation energy transfer from the 2D CHP to QDs, generating spin‐polarized excitons. Interface coupling between CHP and QDs extended exciton spin lifetimes from 3.4 to 14.4 ps, suppressing spin decoherence and amplifying CPL emission. This work ingeniously repurposes the inherent non‐radiative recombination of 2D CHPs, traditionally a drawback, into a spin‐control mechanism that enhances the CPL efficiency of QDs. By elucidating the roles of energy transfer and interfacial spin manipulation, the study not only broadens the optoelectronic applicability of CHPs but also establishes a blueprint for high‐performance CPL‐active devices.

Rogach et al. pioneered a breakthrough strategy to circumvent the intrinsic charge transfer inefficiencies plaguing conventional low‐dimensional CHPs. By implementing in‐situ chiral ligand modification of CsPbBr_3_ NCs with R/S‐β‐PEABr molecules, they simultaneously achieved a near‐unity photoluminescence quantum yield (PLQY ≈ 89%) and robust spin‐polarized emission.^[^ ^172^ ^]^ This approach fundamentally eliminates the requirement for discrete spin‐filtering components by strategically utilizing chiral nanocrystalline assemblies with intrinsic spin‐filtering capabilities as multifunctional emissive media, streamlining device architecture to simultaneously boost interfacial charge injection kinetics. Further optimization was realized through a dual‐hole transport layer (poly‐TPD/PBGC), which minimizes electron leakage and optimizes carrier balance. The resulting spin‐polarized light‐emitting diodes (spin‐LEDs) deliver record‐setting performance with a luminance of 12 800 cd m^−2^, an external quantum efficiency (EQE) of 15.4%, and a CP‐EL dissymmetry factor (g_CP‐EL_) of 2.16 × 10^−3^. The device's efficiency and simplified architecture mark a critical step toward practical spin‐LEDs applications in secure optical communication, quantum light sources, and energy‐efficient polarized displays. Unifying chiral photonic control with perovskite optoelectronics, this strategy opens avenues for the next‐generation spin devices that merge high efficiency with multifunctional light manipulation.

Beard et al. successfully realized an RT spin‐LEDs operating without external magnetic fields by harnessing the CISS effect in CHPs.^[^ ^45^ ^]^ Utilizing the self‐assembled structure of (R/S‐MBA)_2_PbI_4_, they achieved a spin‐polarized hole current exceeding 80%. In the constructed spin‐LEDs (Figure 11d), the chiral organic molecules are oriented perpendicular to the substrate, with their helical axes aligned with the charge transport direction. This configuration selectively filters charge carriers of specific spin orientations through the CISS effect, eliminating the reliance of traditional spin‐LEDs on ferromagnetic materials or magnetic fields. The device could emit CPL at RT (Figure 11e), achieving a polarization degree of ±2.6% at 688 nm, the highest reported value for pure CsPbI_3_ QDs systems. The mechanism arises from the radiative recombination of spin‐polarized holes and unpolarized electrons in the QDs layer, while interface coupling suppresses spin relaxation, extending the spin lifetime to 14 ps and significantly enhancing the CP‐EL efficiency. Electrically driven halide exchange (e.g., Br^−^/I^−^ dynamic substitution) could continuously tune the emission wavelengths from red (688 nm) to green (515 nm). Mixed‐halide QDs (CsPbBr_3x_I_3(1−x)_) exhibit a spin coherence lifetime of 14 ps, a 3.5‐fold increase over pure CsPbI₃ QDs (4 ps), validating spin dynamics optimization through compositional engineering. This work represents a profound integration of spintronics and CHPs optoelectronics, offering a foundation for advancing spin‐photonics.

In parallel, Wang et al. proposed a spin‐LED with ITO/PEDOT: PSS/TFB/CHP/QDs/ZnO/Ag structure, where 2D CHP as a spin‐selective charge transport layer and CdSe/ZnS QDs acts as the emissive layer.^[^ ^173^ ^]^ The optimized device realized a maximum brightness of 5638 cd m^−2^ and an EQE of 2.7%, lower than traditional QDs LEDs (EQE 14.8%), but achieved a g_CP‐EL_ of 1.6 × 10^−2^, confirming effective spin‐polarized emission. Increasing the CHP layer thickness degraded device performance (EQE <1%) but improved g_CP‐EL_, revealing a trade‐off between efficiency and polarization. The possible reasons for the performance degradation were analyzed. The hole mobility of CHPs is significantly lower than that of the ETL, exacerbating the imbalance in carrier recombination. The high refractive index of perovskites (n ≈ 2.4) triggers waveguide modes, leading to optical losses. Besides, the roughness (3.4–13.6 nm) and interface defects of CHPs affect carrier transport. To address these challenges, strategies such as, designing CHPs with high spin polarization and PL efficiency, reducing non‐radiative recombination via core‐shell QDs structures, and extending QDs spin lifetimes using strong SOC materials, are proposed. Despite current efficiency limitations, the device's magnetic‐free operation at RT and clear optimization pathways underscore its potential for applications in displays and encrypted communications.

Wang et al. further investigated the carrier dynamic of CHP/CdSe‐ZnS QDs composites for optimizing the luminescence.^[^ ^174^ ^]^ Time‐resolved photoluminescence (TR‐PL) revealed a shortened fluorescence lifetime in the composite (3.08 ns vs. 8.22 ns for pristine QDs), attributed to hole injection from CHPs into QDs, which introduces non‐radiative recombination pathways. Time‐resolved absorption spectroscopy (TAS) confirmed the charge transfer from CHPs to QDs, emphasizing the critical role of spin‐polarized carrier recombination in enhancing CPL activity. This study systematically correlates CHP lattice distortions with optical activity and proposes a novel strategy to amplify QD CPL through the CISS effect. However, the PLQY of QDs decreased from 40% to 30% in the composite, highlighting the need for interface engineering to mitigate efficiency losses.

Zhang et al. achieved high‐performance sky‐blue spin‐LEDs through the innovative device architecture (ITO/PEDOT: PSS/CHP/TPBi/LiF/Al), where chiral ionic liquids (CILs) play a dual role in defect passivation and chiral induction.^[^ ^171^ ^]^ By coordinating the C = O groups of CILs with Pb^2+^ ions in the perovskite lattice, the defect state density was significantly reduced from 8.1 × 10^18^ cm^−3^ to 4.7 × 10^18^ cm^−3^, enhancing the PLQY (>80%) and extending the fluorescence lifetime from 3.0 to 16.5 ns. Simultaneously, the chiral amino groups in CILs induced the formation of chiral superstructures within the perovskite, strengthening chiral‐induced SOC and spin‐polarized charge carrier generation. Energy level alignment analysis (Figure 11f) confirmed compatibility between the CHP layer, TPBi, and PEDOT: PSS, facilitating efficient charge separation and injection. The resulting device emits sky‐blue CP‐EL at 491 nm (Figure 11g), achieving an EQE of 13.0% and g_CPL‐EL_ of 0.158, the highest reported value for blue spin‐LEDs. Electrical characterization (Figure 11h) revealed a low turn‐on voltage of 3 V, with current density rising exponentially to 10–20 mA cm^−2^ in the 5–6 V range, accompanied by a peak brightness exceeding 1500 cd m^−2^. These metrics underscore the improved carrier injection efficiency enabled by the CILs‐modified perovskite layer. This study pioneers efficient sky‐blue perovskite spin‐LEDs through a chiral‐defect synergistic regulation strategy, elucidating the dual functionality of CILs in defect passivation and chiral‐to‐spin conversion. By harmonizing material design with device engineering, the work establishes a novel paradigm for multifunctional optoelectronic systems, advancing the development of high‐efficiency and polarization‐tunable light sources for applications in quantum optics and full‐color displays.

### 4.4 Circularly Polarized ASE and Lasing

For halide perovskite, lasing is an attractive application and represents a milestone in modern photonics. Notably, CHPs inherit multiple advantages from halide perovskite and feature chirality characteristics. The unique structural and chiroptical properties position CHPs as frontrunners in advancing chiral lasing, a technology that generates coherent CPL with controlled spin states. Intrinsic properties of CHPs, such as strong SOC, exceptional optical gain, tunable bandgap, and spin‐dependent amplification, are advantageous in building chiral lasing systems. Moreover, the chiral crystal lattice enhances spin‐polarized carrier recombination and directly couples photon spin to material chirality, generating CPL lasing without post‐emission filtering. This eliminates the need for external polarizers or complex optical setups, simplifying device design. Additionally, efficient light emission of CHPs reduces lasing thresholds, and compositional engineering (e.g., adjusting halides like Br, I, or Cl) allows wavelength‐specific laser applications.

**Figure 12:**
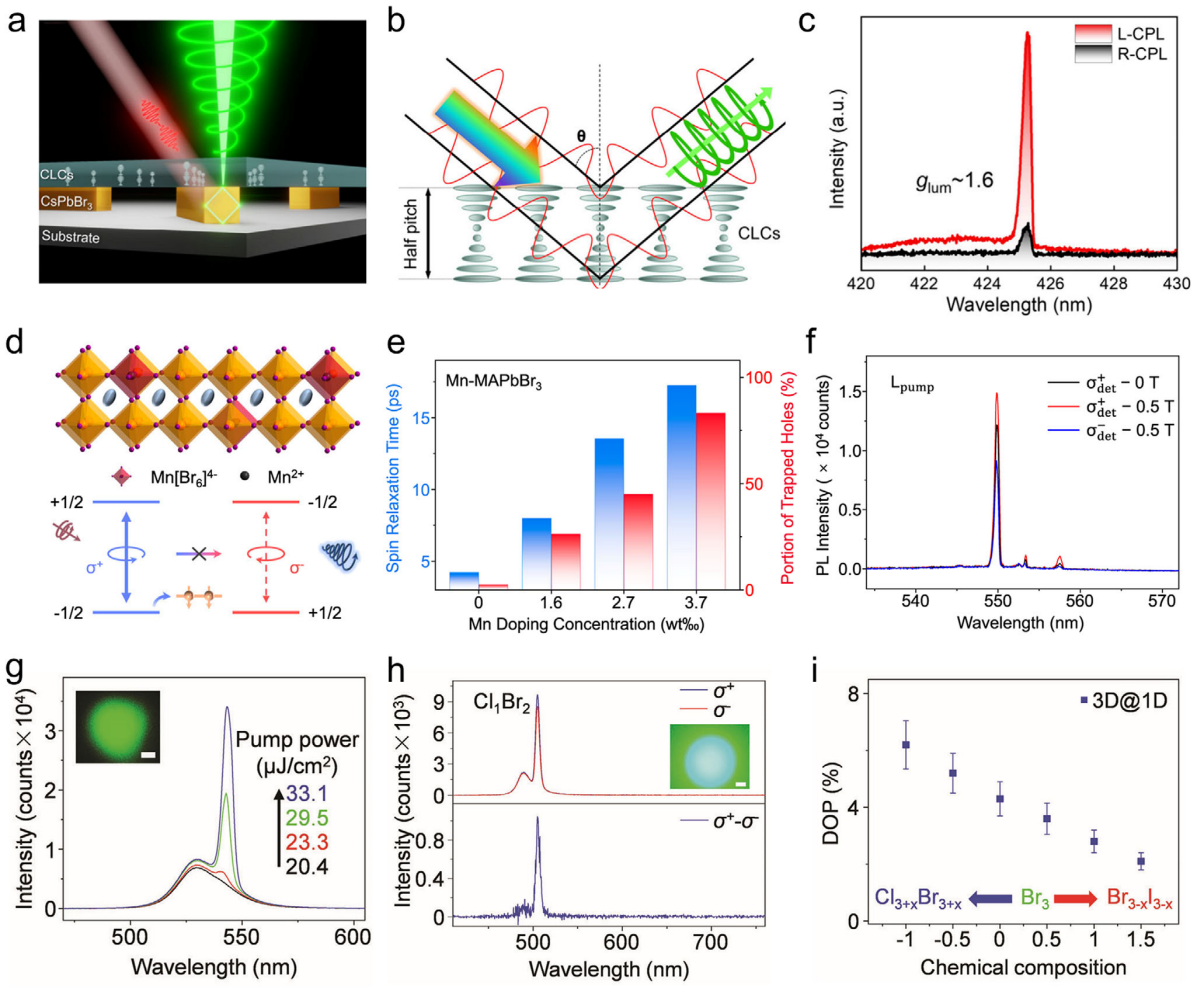
Chiral lasing. a) Sketch of chiral microlaser device. b) Bragg reflection of CLCs. c) CPL lasing. Reproduced with permission.^[^ ^175^ ^]^ Copyright 2024, American Chemical Society. d) Sketch diagram exhibiting suppression of spin relaxation by Mn^2+^ doping. e) Effect from Mn‐doping concentration. f) Laser emission under magnetic field. Reproduced with permission.^[^ ^176^ ^]^ Copyright 2024, Nature Publishing Group. g) Power‐dependent PL. h) Polarization‐dependent ASE spectra. i) DOP of halide perovskite with different composition. Reproduced with permission.^[^ ^177^ ^]^ Copyright 2023, Wiley‐VCH.

Currently, different strategies including doping, chiral metasurface coupling, liquid crystal insertion have been proposed to gain chiral lasing, but most cases are based on halide perovskite.^[^ ^178^, ^179^, ^180^, ^181^ ^]^ To attain chiral lasing, Jiang et al. prepared CsPbCl_x_Br_3−x_ MW using a one‐step rapid anti‐solvent induced recrystallization method.^[^ ^175^ ^]^ The rectangular cross‐section of halide perovskite MW supports whispering gallery mode (WGM) microcavities, attaining a Q factor of up to 3017 and enabling low‐threshold (81.8 µJ cm^−2^) single‐mode lasing. Further, the MW is assembled with the encapsulated cholesteric liquid crystal (CLC) layer (**Figure** **12**a) for achieving chiral lasing. By utilizing the helical photonic crystal structure of CLC, a linearly polarized laser can be converted to a circularly polarized laser through selective Bragg reflection (Figure 12b). Adjusting the photonic bandgap (PBG) of CLC to match the emission wavelength of halide perovskites, a significant enhancement in the dissymmetry factor (g_lum_≈1.62) is achieved, as shown in Figure 12c. This work leverages the high quality (Q) factor of WGM microcavities and the selective reflection mechanism of CLC to establish a new paradigm for chiral lasing devices. Meanwhile, it addresses key challenges in chiral laser technology, such as high gain, tunability, and stability, through the innovative combination of perovskite MW with CLC layers. Under high pump conditions generated by lasers, the electron‐hole exchange interaction (Bir‐Aronov‐Pikus, BAP mechanism) becomes the dominant factor in spin relaxation in perovskites, making it difficult to maintain spin polarization. Zhao et al. utilized Mn^2+^ doping to suppress the electron‐hole exchange interaction (Figure 12d), achieving spin‐polarized laser emission for the first time in halide perovskites with a circular polarization degree (DCP) of 27.3%.^[^ ^176^ ^]^ TA measurement on the MAPbBr_3_ with different Mn concentrations was performed to study the inside carrier dynamics. With the increasing concentration of Mn (Figure 12e), both the trapped hole and spin relaxation time increase, indicating that the enhanced spin relaxation time arises from the suppression of the electron‐hole exchange interaction by trap levels through hole capture. The improved spin lifetime promotes the generation of spin‐polarized lasing. Moreover, utilizing the magnetism of Mn^2+^, the CPL laser emission can be modulated by an external magnetic field (Figure 12f), and DCP (10%–40%) can be dynamically regulated through a magnetic field (±0.5 T). This work reveals the photophysical mechanisms of spin relaxation in perovskites and proposes a universal ion doping strategy (such as Mn^2+^, Co^2+^, etc.) to regulate spin properties for obtaining chiral lasing.

Liu et al. designed a 1D‐anchoring 3D perovskite composite structure, utilizing excitonic chirality transfer from chiral 1D perovskites to non‐chiral 3D perovskites to directly generate spin‐polarized excited states within the gain medium, eliminating the need for external spin injection.^[^ ^177^ ^]^ The CD signal from the chiral 1D perovskite is transferred to the 3D perovskite, which is intrinsically non‐chiral, yet generates circularly polarized luminescence through excitonic chirality transfer. When the pump power exceeds the threshold (29.5 µJ cm^−2^), a narrow emission peak emerges (FWHM ≈ 5.8 nm), confirming amplified spontaneous emission (ASE) behavior (Figure 12g). By tuning the halide composition ratio (e.g., Cl/Br, Br/I), the ASE wavelength could be modulated across 510–610 nm, and Figure 12h shows the ASE at 510 nm. Despite tunable emission wavelength through composition engineering, Cl doping reduces the SOC strength and prolongs the spin relaxation time. A maximum DOP of 6.2% (Figure 12i) is achieved among 1D‐anchoring 3D perovskites with diverse halide compositions. This work demonstrates, for the first time, self‐triggered spin‐polarized lasing based on excitonic chirality transfer through an innovative 1D‐anchoring 3D perovskite design, offering a novel strategy for chiral lasers.

Direct chiral perovskite ASE could be seen as a promising pathway toward low‐threshold, spin‐polarized lasing due to its intrinsic chirality and high optical gain. The fabrication of CHP nanostructures further enhances this potential by leveraging advanced resonators (optical cavities), such as WGM, Fabry‐Perot (FP), distributed feedback (DFB), and bound in continuum state (BIC) configurations, to achieve efficient light confinement and feedback. For example, WGM cavities exploit the ultrahigh Q factors of CHP microdisks or microspheres to enable ultralow threshold lasing with circularly polarized outputs. FP cavities allow wavelength‐tunable chiral lasing through cavity‐length modulation, though requiring additional chiral mirrors for polarization selectivity. DFB cavities integrate periodic nanostructures (e.g., chiral gratings) to directly couple CHP's spin‐dependent gain with resonant modes, achieving high polarization purity. Despite these theoretical advantages, experimental demonstrations of CHP‐based chiral lasers remain limited. Key challenges include optimizing material stability under optical/electrical pumping, enhancing the dissymmetry factor of ASE, and achieving scalable integration of chiral nanostructures with photonic cavities.

### 4.5 Chiral Second Harmonic Generation of CHPs

SHG technology with frequency conversion and polarization sensitivity features finds widespread applications in various fields such as imaging, communication, and energy. The generation of SHG depends on materials with a non‐centrosymmetric crystal structure. CHPs fulfill the requirement by incorporating chiral organic molecules, such as R/S‐MBA or chiral amines, which disrupt the lattice symmetry. This disruption induces helical or layered chiral arrangements that satisfy the phase‐matching conditions essential for efficient SHG. A synergistic interaction between these chiral ligands and inorganic frameworks, such as lead halide [PbX_6_]^4−^ octahedra, enhances the asymmetric distribution of electron clouds, thereby amplifying the second‐order nonlinear polarization. Furthermore, chiral structures endorse circularly polarized second harmonic generation (CP‐SHG), where the asymmetry factor is determined by the spatial arrangement of chiral molecules and their exciton‐photon coupling strength. This phenomenon unlocks novel opportunities in advanced applications like chiroptical encoding and quantum communication technologies.

**Figure 13:**
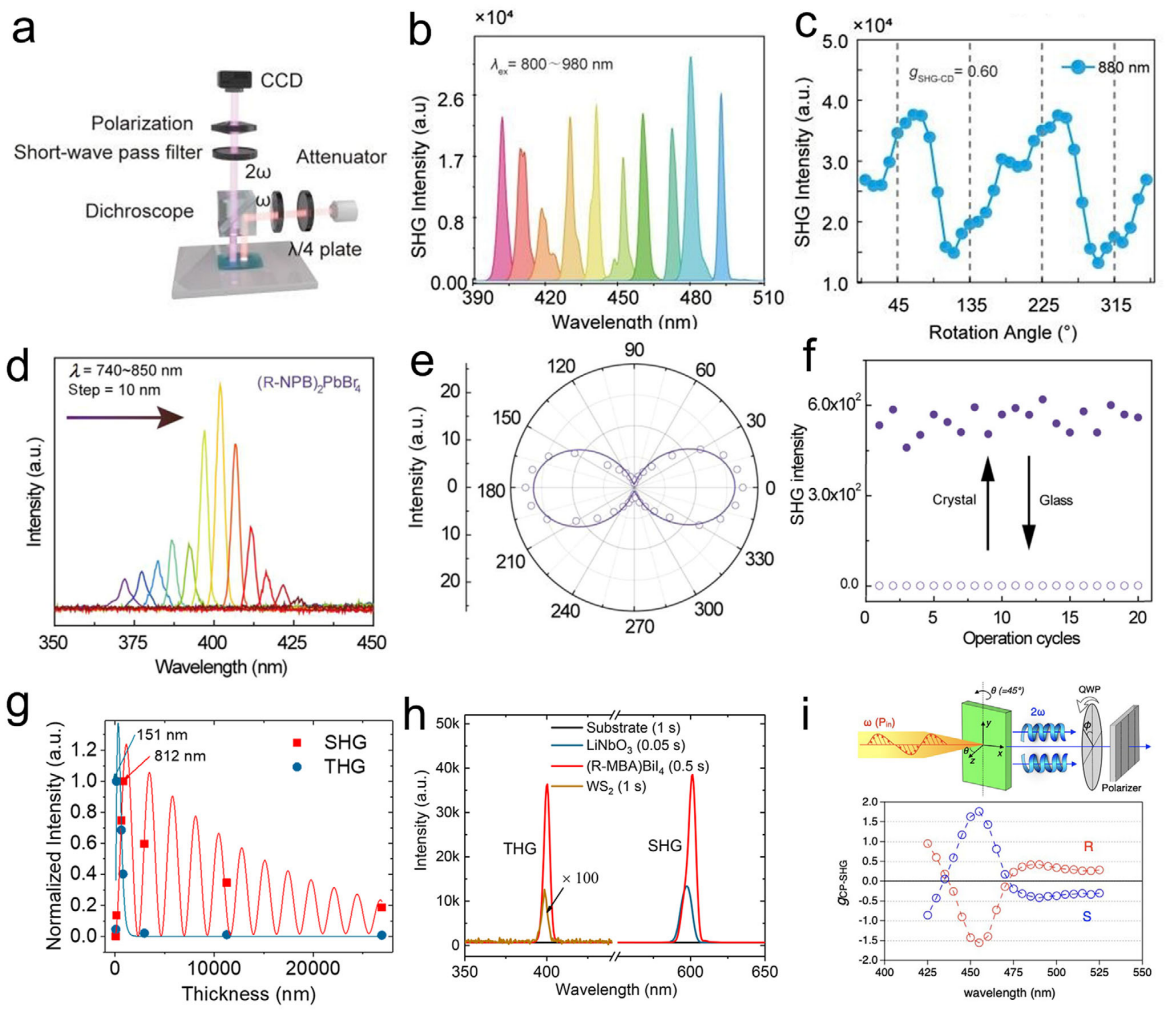
Emerging SHG application based on CHPs. a) Micro‐area NLO characterization system. b) SHG intensity of CHP at different wavelengths. c) The relationship between CP‐SHG intensity and quarter‐wave plate rotation angle. Reproduced with permission.^[^ ^182^ ^]^ Copyright 2024, Wiley‐VCH. d) SHG intensity of CHP under excitation wavelengths ranging from 740 to 850 nm. e) The relationship between SHG intensity and wavelength. f) Switchable SHG between amorphous and crystalline states of R‐type CHP. Reproduced with permission.^[^ ^183^ ^]^ Copyright 2022, Wiley‐VCH. g) SHG and THG response for CHP with different thicknesses. h) SHG and THG of WS_2_, CHP, and LiNbO_3_. Reproduced with permission.^[^ ^184^ ^]^ Copyright 2021, American Chemical Society. i) Setup for measurement of CP‐SHG (up panel) and g_CP‐SHG_ of CHPs. Reproduced with permission.^[^ ^48^ ^]^ Copyright 2024, Wiley‐VCH.

Long and coworkers pioneered the first chiral non‐metallic perovskite, (R/S‐MBA)_6_Cl(NH_4_Cl_6_), where chirality arises from the helical stacking of α‐methylbenzyl ammonium (MBA⁺).^[^ ^182^ ^]^ This inverse perovskite architecture, featuring anionic A/B sites and a cationic X site, overcomes limitations inherent to conventional perovskites such as metal ion toxicity, high production costs, and low laser damage thresholds (LDTs). A custom‐built micro‐area NLO characterization system employing reflective collection mode (**Figure** **13**a) revealed a broadband SHG response across 800–980 nm excitation wavelengths (Figure 13b), achieving a second‐order nonlinear coefficient (deff) of 0.89 pm V^−1^. This performance exceeds commercial Y‐cut quartz (0.3–0.5 pm V^−1^) and numerous Cd/Cu‐containing systems. This material demonstrates remarkable SHG CD asymmetry (g_SHG‐CD_ = 0.60, Figure 13c), signifying pronounced nonlinear chiroptical activity suitable for polarization modulation and chiral sensing. The measured LDT of 71 mJ cm^−2^ significantly exceeds values reported for conventional CHPs, a performance enhancement fundamentally rooted in the material's broadband optical transparency spanning 300–1000 nm spectrum combined with its organic constituents’ minimal photon absorption characteristics. These inherent material properties collectively establish its operational viability for high‐power laser and quantum optics applications.

Fu et al. synthesized 1D CHP single crystal [(R/S)‐3‐aminopyridine]PbI₄ (1R/1S), crystallizing in space group *P2_1_2_1_2_1_*.^[^ ^185^ ^]^ Hydrogen bonding induces a helical arrangement of [PbI_6_]^2^⁻ octahedra (Δd ≈ 2 × 10⁻^3^), breaking mirror symmetry to attain chiroptical responses. When LCP/RCP propagates in a non‐centrosymmetric crystal, the difference in refractive indices leads to varying SHG efficiency, manifested as the anisotropy factor g_SHG‐CD_. The SHG intensity is 2.1 times that of KH_2_PO_4_ (KDP), outperforming the most well‐known hybrid perovskite (0.4–0.9 times KDP). Besides, this CHP crystal exhibits high near‐infrared transparency of 80%, excellent thermal stability (>550 K), and humidity stability (45% RH for 30 days). Zhao et al. utilized a CBA strategy to fabricate single‐crystal MW arrays with pure (001) orientation and defect‐free morphology for SHG.^[^ ^183^ ^]^ These arrays manifest an order‐of‐magnitude SHG enhancement compared to spin‐coated films. Wavelength and polarization‐dependent SHG behaviors were investigated. Within the excitation wavelength range of 740–850 nm, the SHG signal shows peaks at 400–425 nm corresponding to half the excitation wavelength (Figure 13d), indicating the nonlinear response capability over a broad spectral range. As the excitation wavelength approaches the absorption edge (390 nm) of CHP, the SHG intensity gradually increases, possibly due to excitonic resonant enhancement within the band structure. The polar plot in Figure 13e showcases that the SHG signal is strongest along the MW axial direction, indicating that the (010) growth direction of the single crystal MW predominantly governs the anisotropy of nonlinear polarization. Notably, a reversible phase transition between the glassy state and crystalline state can be achieved by modulating the crystal kinetics of perovskites using chiral cations (such as R/S‐NPB). During the phase transition process, no material degradation was observed, and the material exhibited excellent thermal stability. This reversible SHG switching shows an endurance of more than 20 times, as shown in Figure 13f. In the crystalline state, the non‐centrosymmetric space group (P_21/C_) and high crystallinity of the MW array resulted in an SHG intensity reaching 0.68 times that of a KDP crystal. In the glassy state, due to short‐range order and structural isotropy, the SHG signal completely disappears, leading to extremely high switching contrast. This study successfully combines the phase transition characteristics of 2D CHP with their NLO properties through innovative material design and assembly strategies, achieving efficient and reversible SHG switching. This achievement provides a crucial technological path for the next generation of tunable photonic devices, particularly excelling in integration and low power consumption.

Yao et al. synthesized (R/S‐MBA)BiI₄, which possesses a helical chain‐like inorganic framework ([BiI₄]⁻), wherein the chiral MBA⁺ cation induces asymmetric distortion of the bismuth halide octahedra through hydrogen bonding, breaking spatial inversion symmetry and supporting SHG activity.^[^ ^184^ ^]^ The lead‐free CHP exhibits strong free excitons, self‐trapped excitons, and discrete energy levels, establishing the energy matching conditions for resonant enhancement. The material possesses broadband absorption ranging from the visible to near infrared, with CD spectra revealing the Cotton effect, confirming successful chirality transfer to the inorganic lattice. The SHG response covers 400–800 nm, while the third harmonic generation (THG) operates across 265–550 nm, rendering the material suitable for multi‐wavelength integrated photonic devices. Both SHG and THG intensities display nonlinear thickness dependence, achieving a maximum at 812 and 151 nm, respectively (Figure 13g). A quantification of SHG and THG was evaluated as shown in Figure 13h. At a pump wavelength of 1550 nm, the nonlinear coefficient (χ^2^) for SHG reaches 130.5 pm V^−1^ (1.6 times that of LiNbO_3_), while the χ^3^ for THG reaches 9.0 × 10^6^ pm^2^ V^−2^ (37 times that of monolayer WS_2_). This breakthrough in SHG/THG performance, achieved through 1D chiral bismuth halide design, establishes resonance enhancement as a novel strategy for optimizing low‐dimensional CHPs as NLO materials.

Yang et al. successfully synthesized the first 0D chiral lead bromide perovskite by incorporating chiral organic cations with dual chirality centers, significantly enhancing structural asymmetry.^[^ ^186^ ^]^ The 0D material exhibits a g_SHG‐CD_ value 2.6 times greater than the 1D counterparts, outperforming analogous Cu/Bi‐based perovskites. This enhancement arises from strong electron‐phonon coupling and low‐dimensional quantum confinement effects. Remarkably, the 0D CHP achieves a LDT of 59.36 GW cm^−2^, far exceeding conventional nonlinear crystals like KDP (≈1 GW cm^−2^), making it viable for high‐power laser systems. This work elucidates the synergistic role of low‐dimensional architectures and electron–phonon interactions in amplifying NLO properties, establishing a new paradigm for designing high‐performance CHP materials and advancing their applications in photonics and quantum technologies. Relying on SHG‐CD techniques, Araoka et al. revealed for the first time the key role of magnetic dipole (MD) transitions in the nonlinear optical response of (R/S‐MBACl)_2_PbI_4_.^[^ ^48^ ^]^ Under linear polarized light excitation, the CHP outputs CP‐SHG, with a maximum g‐factor of 1.76 at 455 nm (Figure 13i), which is among the highest values reported for chiral materials to date. The work reveals the decisive role of MD transitions in the NLO chirality of 2D CHP through precise material design and innovative experimental methods, opening new avenues for efficient CPL manipulation.

The SHG is a cornerstone technology in the fields of laser, optical communications, and biological imaging. Traditional inorganic NLO materials (such as LiNbO₃, KDP) exhibit excellent performance, but their complex preparation and integration difficulties limit their application. CHPs, with their non‐centrosymmetric structures, strong nonlinear responses, and polarization tunability, have been popular materials for SHG. The convergence of chiral engineering and micro‐nano photonics holds promise for breakthroughs in high‐resolution imaging, quantum light sources, and on‐chip optical interconnects. In the future, synthesis processes should be optimized to enhance the stability of CHP. Meanwhile, the effect of dimensionality of CHPs on the nonlinear responses deserves exploration.

### 4.6 CHP‐Spintronic‐Devices

In chiral materials, the spin orientation of electrons becomes coupled to molecular helicity during transport processes, leading to preferential transmission of electrons with specific spin states (left‐ or right‐handed) and the generation of spin‐polarized currents. This phenomenon originates from SOC, which is significantly amplified by the structural asymmetry inherent to chiral systems. CHPs exemplify this principle through their hybrid architecture, where chiral organic cations (R/S‐MBA⁺) are integrated with inorganic frameworks such as [PbI_6_]^4−^ octahedra. The resulting non‐centrosymmetric crystal structure breaks spatial inversion symmetry, thereby intensifying SOC effects. Recent studies report spin polarization degrees exceeding 80% in CHP thin films, a substantial improvement over conventional achiral materials. Spintronic applications depend critically on precise control of electron spin states, necessitating materials capable of efficient spin injection, transport, and detection. The CISS effect observed in CHPs provides a novel mechanism for achieving spin polarization without requiring external magnetic fields. Furthermore, CHPs can be processed via solution‐based methods, which are compatible with flexible electronics and large‐scale production.

**Figure 14:**
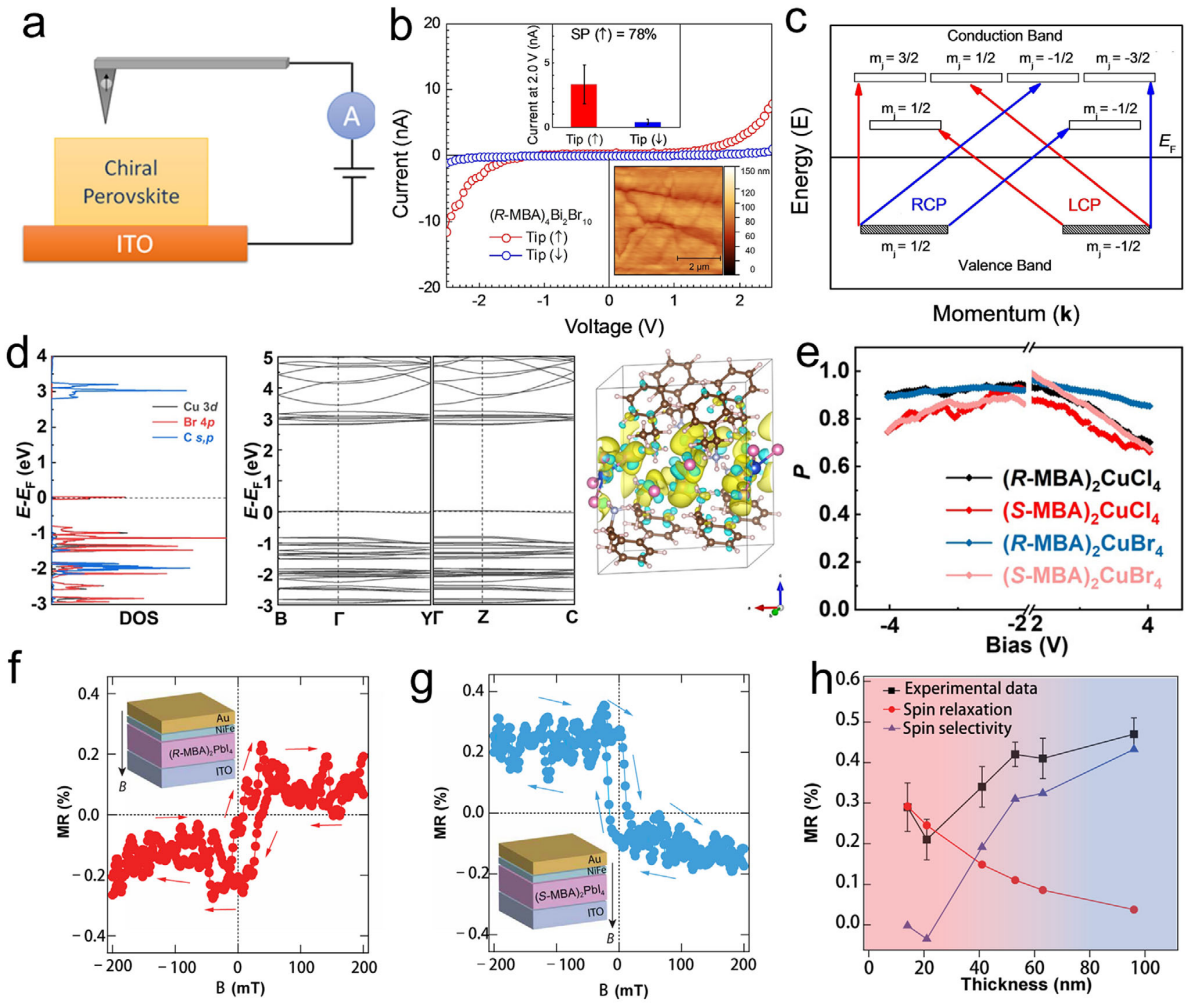
Spintronics. a) Schematic of the mc‐AFM measurement of CHP. b) Current response of CHP/ITO under different tip magnetization. c) Optical selection rule under the illumination of CPL. Reproduced with permission.^[^ ^187^ ^]^ Copyright 2022, Wiley‐VCH. d) DFT calculation of (R‐MBA)_2_CuBr_4,_ including projected density of states (left), band structure (middle), and differential charge density (right). e) Spin polarization degree of (R/S‐MBA)_2_CuCl_4_ and (R‐MBA)_2_CuBr_4_. Reproduced with permission.^[^ ^188^ ^]^ Copyright 2021, Wiley‐VCH. f). MR response of (R‐MBA)_2_PbI_4_. g) MR response of (S‐MBA)_2_PbI_4_. h) Thickness‐dependent MR response. Reproduced with permission.^[^ ^189^ ^]^ Copyright 2019, AAAS.

To investigate the magneto‐optical activities in CHP, Maiti et al. synthesized (R/S‐MBA)_4_Bi_4_Br_10_, where the inorganic [Bi_2_Br_10_]^4−^ dimer coordinates with chiral organic cations to form a non‐centrosymmetric 0D structure (space group P2_1_).^[^ ^187^ ^]^ The dimension increases the density of chiral ligands, thereby boosting the CISS effect. Substituting bismuth for lead reduces environmental toxicity while retaining SOC. The synergistic effect of CISS and the optical selection rule was inspected for spin carrier dynamics. Vertical transport measurements by mc‐AFM (**Figure** **14**a) validate spin‐selective behavior: R‐perovskite exhibits higher current under an up‐magnetized probe (spin polarization degree = 78%, Figure 14b), while S‐perovskite shows the opposite trend (84% polarization), directly confirming chirality‐dependent spin filtering. According to the optical selection rule (Figure 14c), LCP transfers angular momentum (Jz = −ℏ) to electrons, exciting 67% spin‐up (m_j_ = +1/2) and 33% spin‐down (mj = −1/2) carriers in the conduction band. Conversely, RCP (Jz = +ℏ) generates 67% spin‐down and 33% spin‐up populations. In this work, relying solely on optically induced spin polarization is not sufficient to achieve efficient CPL detection. CISS enhances the anisotropy through the following steps. Optical selection rules ensure the generation of charge carriers with different spin polarizations under CPL irradiation. CHP acts as a spin filter, permitting only charge carriers with specific spin orientations (such as R‐type CHP transmitting spin‐up and S‐type CHP transmitting spin‐down) to pass through. This mechanism lays a theoretical foundation for the development of efficient and eco‐friendly spin‐optoelectronic devices.

Existing CHPs containing lead suffer from high toxicity and poor stability. Copper‐based CHPs offer a promising alternative due to low toxicity and robust magneto‐optical properties. Song et al. synthesized (R/S‐MBA)_2_CuX_4_ (X = Cl, Br), where [CuX_4_]^2−^ adopts a distorted tetrahedral structure.^[^ ^188^ ^]^ Chiral organic cations (MBA⁺) are coupled to the inorganic framework via hydrogen bonds, forming a non‐centrosymmetric monoclinic crystal system (space group *C2*). The bromide (R/S‐MBA)_2_CuBr_4_ undergoes a phase transition from an orthorhombic to a monoclinic crystal system during thin film preparation, whereas the chloride structure remains stable. DFT calculations (Figure 14d) reveal hybridization between Cu *3d* and halogen *p* orbitals near the Fermi level, enhancing chiral charge transport. The R‐enantiomer preferentially transports spin‐up electrons, while the S‐enantiomer does the opposite; the racemic mixture shows no spin selectivity. A spin polarization degree of P = 90% (±2 V) in vertical transport was obtained, surpassing lead‐based perovskites (Figure 14e). This research achieved a lead‐free and highly stable spin‐filtering material by designing 0D chiral copper halides.

Lu et al. measured the vertical charge transport of 2D CHP (R/S‐MBA)_2_PbI_4_ using mc‐AFM and found a significant spin dependence, with a spin polarization degree as high as 86%, surpassing traditional chiral monolayer molecular systems (typically 30–50%).^[^ ^189^ ^]^ Helical potential barriers from R/S‐MBA create spin‐polarized tunneling channels through inorganic‐organic layered stacking, boosting spin selectivity. MR, defined as $MR=\frac{R(B)-R(0)}{R(0)}\times \;100\%$, where R(B) is the device resistance under magnetic field and R(0) is the device resistance under zero magnetic field, exhibits chirality‐dependent responses. For (R‐MBA)_2_PbI_4_ (Figure 14f), MR synchronizes with the ferromagnetic electrode's hysteresis loop, showing positive MR (resistance increases with field). The (S‐MBA)_2_PbI_4_ (Figure 14g) displays negative MR (resistance decreases with field). In contrast, non‐chiral (PEA)_2_PbI_4_ exhibits only weak anisotropic magnetoresistance (AMR), intrinsic to the electrode. CISS‐induced resistance differences arise when spin‐polarized carriers traverse the CHP layer, with reversed chirality inverting spin polarization. Further, the thickness‐dependent MR response was studied as shown in Figure 14h. When the thickness of the CHP layer increases from 28 to 75 nm, the maximum MR value (MR_max_) shows only slight fluctuations. Since the carriers predominantly tunnel through the inorganic‐organic alternate layers in a spin valve device, which helps in avoiding the thickness‐sensitive dependence on spin relaxation traditionally seen in spin valves. Through chiral‐dependent MR sign reversal, weak thickness dependence, and comparisons with conventional devices, this work validates the high efficiency and uniqueness of multilayer chiral structures in spin filtering, providing a crucial experimental basis for developing solution‐processed and energy‐efficient spintronics.

### 4.7 Memory using CHPs

CHPs exhibit intrinsic ferroelectric properties arising from their non‐centrosymmetric crystal structures. The incorporation of chiral ligands induces structural distortions within the inorganic framework, generating spontaneous polarization. This distortion is synergistically amplified by asymmetric hydrogen bonding and steric hindrance effects inherent to the chiral ligands, thereby enhancing ferroelectric polarization intensity. These characteristics make CHPs suitable for high‐durability ferroelectric random‐access memory (FeRAM) applications. By correlating the helical orientation of the chiral structure (R/S configuration) with the polarization direction, multilevel data storage states (e.g., 00, 01, 10, 11) can be achieved, significantly increasing storage density. Furthermore, polarization reversal in CHPs operates at low energy consumption, with driving voltages as low as ≈1 V, positioning them as promising candidates for low‐power non‐volatile memory technologies. Additionally, the coupling of ferroelectric polarization with optical responses in CHPs carries out optically modulated memory functionality, showcasing significant potential for the development of hybrid optoelectronic memory devices.

Recent breakthroughs in hybrid perovskite ferroelectrics highlight remarkable progress in integrating chirality, ferroelectricity, and optoelectronic functionalities. The groundbreaking study by Xiong et al. introduces a novel class of metal‐free three‐dimensional CHP ferroelectrics, exemplified by MDABCO‐NH_4_I_3​_, which combines the structural advantages of perovskites with chirality‐driven functionalities.^[^ ^192^ ^]^ By replacing conventional metal cations with chiral organic molecules (e.g., R/S‐3AP^2+^ or R/S‐3AQ^2+^), the authors synthesized enantiomorphic perovskites crystallizing in polar space groups (*P*2_1_), where the intrinsic chirality of the organic cations induces symmetry breaking and spontaneous polarization. These CHPs exhibit eight switchable polarization directions, enabled by the alignment of molecular dipoles and structural distortions at both A‐ and B‐sites, achieving a spontaneous polarization (*P_s_*) of 22 µC cm^−2^ and a high phase transition temperature (T_0_) of 448 K. The chirality of the organic components not only drives the formation of polar crystal structures but also introduces optical activity, as demonstrated by mirror‐image vibrational circular dichroism (VCD) spectra for enantiomeric pairs (e.g., R‐3AP‐NH_4_Br_3_ versus S‐3AP‐NH_4_Br_3_). This optical asymmetry, coupled with selective responses to CPL, positions CHPs as promising candidates for spin‐selective electronics and optical data storage, where handedness‐dependent light‐matter interactions could spark novel read/write mechanisms. Furthermore, the materials’ multi‐axial polarization and low coercive fields (≈6–12 kV cm^−1^) enhance their potential for high‐density non‐volatile memory applications, allowing each unit cell to encode multiple bits of information. Despite these advances, challenges remain in optimizing long‐term stability against organic degradation and scaling up synthesis via methods like chemical vapor deposition (CVD). Future efforts to integrate CHPs with 2D materials or engineer robust hybrid architectures could unlock their full potential in flexible electronics, bio‐compatible devices, and energy‐efficient photonic systems, bridging the gap between ferroelectricity, chirality, and quantum technologies.

**Figure 15:**
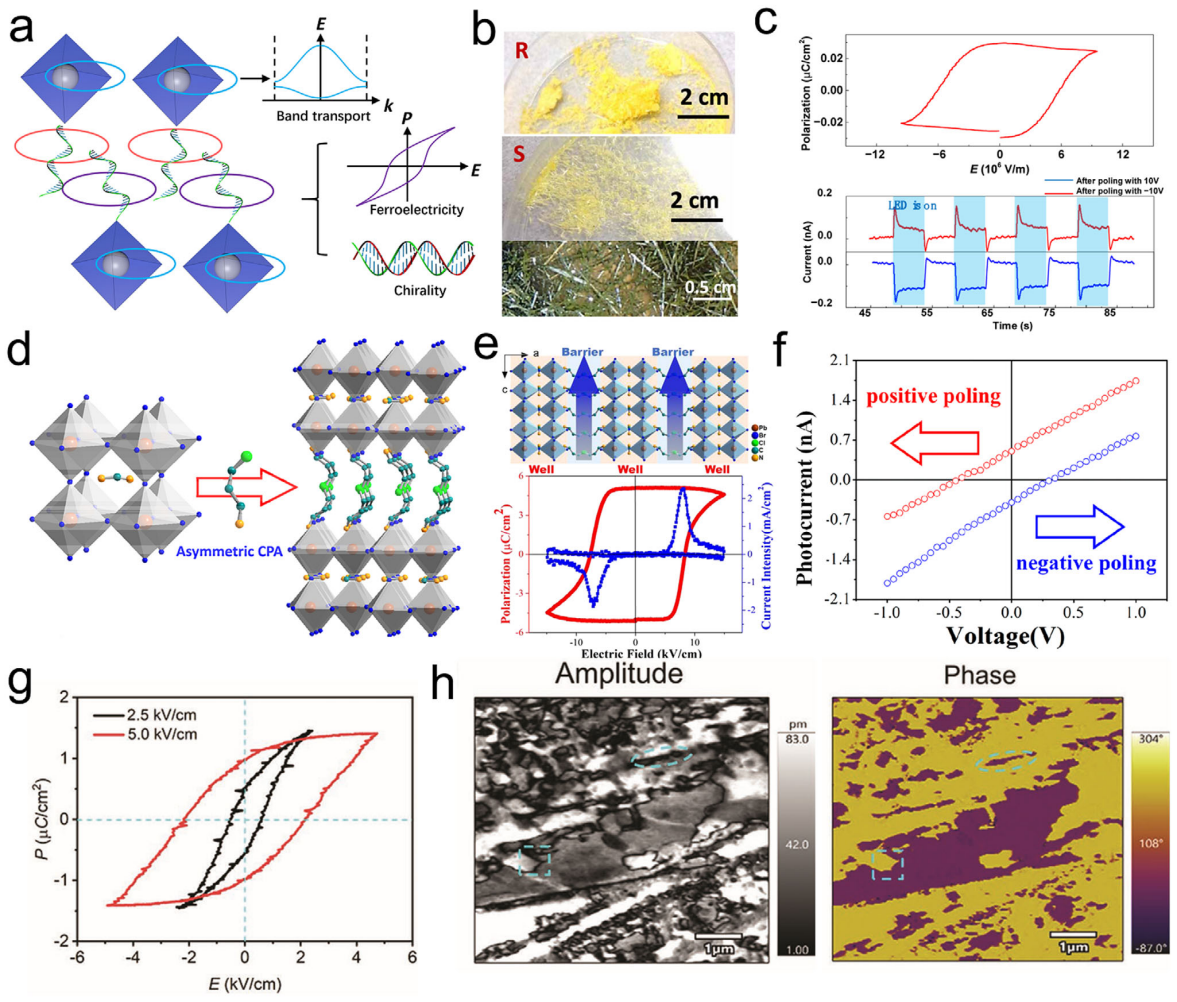
Memory on ferroelectric properties. a) CHP design for achieving semiconducting property, ferroelectricity, and chirality. b) Optical image of the synthesized CHP. c) *P‐E* characteristics (up panel) and switchable diode effect (down panel). Reproduced with permission.^[^ ^44^ ^]^ Copyright 2020, AAAS. d) Photoferroelectric CHP obtained by the introduction of CPA. e) *P‐E* loop and current intensity versus electric field. f) I‐V characteristics measured under positive and negative pooling. Reproduced with permission.^[^ ^190^ ^]^ Copyright 2022, American Chemical Society. g) P−E loops measured at 298 K. h) PFM measurement of in‐plane domain structure. Reproduced with permission.^[^ ^191^ ^]^ Copyright 2020, Wiley‐VCH.

Shi et al. successfully synthesized novel low‐dimensional CHPs (R/S‐CYHEAPbI₃), which for the first time combines the chirality, ferroelectricity, and switchable photovoltaic effect.^[^ ^44^ ^]^ This material features a semiconductor‐active chain of PbI_6_ octahedra and chirality arranged cyclohexyl ethylamine cations, causing symmetry breaking and polarization (**Figure** **15**a). Millimeter‐sized single crystals (Figure 15b) and thin films were synthesized using solution‐based methods and CVD, respectively. Further, DFT calculations corroborate its indirect bandgap, Rashba‐Dresselhaus SOC, and polarization along the b‐axis. Experimental validation via CD spectroscopy revealed optical activity exceeding conventional chiral organic compounds. Ferroelectricity was confirmed by polarization‐electric field (*P‐E*) hysteresis loops (Figure 15c, top), pyroelectric effects, and temperature‐dependent dielectric constants, with a Curie temperature of ≈100 °C. Polarization intensities ranged from 0.03 to 1.2 µC/cm^2^, lower than traditional ferroelectrics like PZT. Moreover, the material exhibited switchable photocurrents under zero bias, enabled by polarization‐dependent band bending (Figure 15c, bottom), suggesting promise for non‐volatile optoelectronic memory.

Liu et al. developed an achiral 2D bilayer ferroelectric crystal (CPA)_2_FAPb_2_Br_7_ (CPA = chloropropylammonium, FA = formamidinium) as shown in Figure 15d.^[^ ^190^ ^]^ This material crystal belongs to the *mm*2 point group (non‐chiral polar point group) and achieves optical activity through symmetry breaking, without the need for chiral ligands. It exhibits a spontaneous polarization intensity of 5.1 µC cm^−2^, and a Curie temperature of 328 K, which were confirmed through polarization‐electric field hysteresis loops (Figure 15e) and phase transition analysis. When the external electric field exceeds the coercive field, ferroelectric domains undergo flipping, resulting in a change in polarization direction. During the polarization reversal process, the rapid change in polarization intensity generates a transient displacement current, manifested as a current peak in the hysteresis loop measurement. This current is used to characterize the polarization‐switching characteristics of ferroelectric materials and forms the physical basis for the operation of FeRAM. The bandgap of the hybrid crystal is 2.72 eV, showing strong UV‐visible light absorption capabilities. By combining ferroelectric polarization with the SOC of photo‐generated charge carriers, this crystal achieved an ultra‐high anisotropy factor. Moreover, the direction of zero‐bias photocurrent reverses after positive/negative polarization (Figure 15f), directly demonstrating the control of current by the polarization direction. Ferroelectric polarization, through its built‐in electric field, carrier spin coupling, and dynamic polarization switching, offers multidimensional control over current generation and direction, which bestows unique advantages on ferroelectrics in non‐volatile memory, quantum sensing, and self‐powered optoelectronic device applications.

Further expanding the scope, Fan et al. synthesized 2D chiral Dion‐Jacobson‐type perovskite ferroelectrics (R/S‐3AMP)PbBr_4_ (3AMP = 3‐aminomethylpyridinium cation), integrating ferroelectricity, chirality, and Rashba‐Dresselhaus effect.^[^ ^191^ ^]^ This CHP exhibits a ferroelectric phase and a paraelectric phase (PEP). For the former, the inorganic layer [PbBr_6_] octahedra are significantly distorted (bond angle deviation Δα = 14°), and the organic cations are orderly arranged, forming spontaneous polarization along the b‐axis. For the latter, the organic cations become disordered at high temperatures (>420 K), and the symmetry of the inorganic layer increases, resulting in a disappearance of the polarization. The CHP exhibits a spontaneous polarization strength of 1.0 µC cm^−2^ and a Curie temperature of 420 K, verified through hysteresis loops (Figure 15g). Further, the piezoresponse force microscopy (PFM) attests that the reproducible switching of domain structure could be modulated by the applied positive and negative bias. The lateral PFM (in‐plane polarization component) reveals irregular domains (ellipses and boxed markers in Figure 15h), while the vertical PFM (out‐of‐plane polarization component) shows a weaker response, indicating that the polarization is primarily oriented along the in‐plane direction. The synergy of ferroelectric polarization and Rashba‐Dresselhaus effects in single‐crystal CHPs enhances photocurrent anisotropy, advancing multiferroic and spintronic applications.

The incorporation of chiral structures in hybrid perovskites plays a pivotal role in lowering lattice symmetry, thereby facilitating the formation of ferroelectric phases. The ordered alignment of chiral organic cations generates intrinsic dipole moments, which synergize with distortions in the inorganic sublattice to amplify ferroelectric polarization. This strong coupling between chiral molecules and inorganic frameworks not only enhances remnant polarization but also leverages chiral hydrogen‐bond networks to suppress ion migration and polarization fatigue, thereby extending the durability of ferroelectric switching cycles. Furthermore, chiral architecture endows these materials with a multifunctional response to external stimuli such as light, electric fields, and magnetic fields. Examples include light‐driven polarization reversal and magnetic‐field‐guided chiral domain wall motion, which open avenues for multidimensional data storage. However, the practical application of CHPs faces challenges, as their organic components are susceptible to racemization under elevated temperatures or large electric fields. Addressing these limitations requires innovative synthetic strategies, such as low‐temperature antisolvent crystallization or template‐assisted growth, to preserve chiral integrity. Concurrently, optimizing the molecular design to improve inorganic‐organic interfacial compatibility and implementing stability‐enhancing optimization, such as surface passivation, encapsulation, or hydrophobic functionalization, are critical for mitigating environmental sensitivity. By harnessing structural symmetry‐breaking and organic‐inorganic synergy, CHPs achieve superior ferroelectric storage performance characterized by high polarization intensity, low energy consumption, and multilevel storage capacity, being promising candidates for next‐generation high‐density and low‐power memory technologies.

## 5 Challenges and Perspectives

CHPs represent a frontier where chiroptics, optoelectronics, and spintronics converge. Despite their remarkable potential, the journey from laboratory‐scale demonstrations to widespread commercial adoption of CHPs is laden with significant hurdles. These challenges span multiple aspects, from material‐specific limitations to device‐level inefficiencies and system‐integration complexities. Addressing these issues requires a multi‐pronged approach that combines fundamental research, innovative engineering, and cross‐disciplinary collaboration. The following perspectives outline key areas of focus for advancing CHP‐based technologies towards practical applications:

1. Material engineering for chiroptical enhancement: Low‐dimensional CHP nanocrystals or QDs currently face the dual problems of low yield and limited CD values. To improve chiroptical performance, researchers could explore novel synthetic routes. For example, the use of template‐assisted synthesis methods might help in precisely controlling the growth of QDs, leading to higher yields and improved chiroptical properties. Additionally, doping with specific elements or molecules that can enhance the chiral interactions within the QDs could be investigated. In the case of bulk 3D CHP films, advanced defect passivation techniques are required. This could involve the development of new passivating agents that can more effectively trap and neutralize defects, reducing dark currents. Regarding moisture degradation, encapsulation strategies need to be further refined. Hybrid encapsulation materials, combining inorganic and organic layers, could provide better protection against moisture ingress while maintaining the optical and electrical properties of the CHP films. As for the scalable fabrication of high‐density 1D NW arrays, simplifying the capillary‐based assembly templates is crucial. One approach could be to use self‐assembling polymers as templates, which are easier to fabricate and remove, and can potentially enable the large‐scale production of polarization‐sensitive devices.
2. Device architecture and multimodal sensing: Current two‐terminal CPL‐sensing detectors and polarization‐sensitive PAS are limited in their ability to integrate multiple types of information. They typically focus on either polarization or basic electrical signals, neglecting the potential to simultaneously detect wavelength and spin information. Future efforts should concentrate on developing photodiode/FET architectures with insert layers that can effectively modulate photo‐induced carriers. These insert layers could be designed to have specific optical or electrical properties that enable the selective detection of different parameters. For example, incorporating materials with wavelength‐dependent absorption coefficients into the insert layers could allow the device to distinguish between different wavelengths of light. Similarly, materials with spin‐related properties could be used to detect spin information. By integrating these capabilities, intelligent sensors could be created that can process multi‐parameter optical inputs, providing a more comprehensive understanding of the light environment. This would be particularly useful in applications such as optical communication, where the ability to simultaneously detect polarization, wavelength, and spin could enhance data transmission rates and security.
3. CPL emission and laser technology advancement: Increasing the circularly polarized electroluminescence dissymmetry factor *g*_CP‐EL_ in CHP‐LEDs is a critical challenge for practical applications. Currently, the relatively low *g*_CP‐EL_ values limit the performance of these devices, making them less competitive compared to traditional LEDs. One approach to addressing this issue is to develop high‐mobility chiral ligands. These ligands could facilitate more efficient charge transport within the device, reducing the likelihood of non‐radiative recombination and enhancing the overall electroluminescence efficiency. Another option is to incorporate core‐shell structures, which can improve the confinement of excitons and carriers, leading to increased *g*_CP‐EL_. Exciton–photon strong coupling and hybrid cavity designs offer great promise for the development of low‐threshold chiral lasers. By combining chiral perovskites with topological or plasmonic structures, it may be possible to achieve dynamic polarization control under ambient conditions. Topological structures can provide unique optical properties, such as robust light propagation and enhanced light‐matter interactions, while plasmonic structures can enhance the local electromagnetic field, facilitating the generation of laser light. Electrical pumping schemes for practical spin‐laser diodes also need to be explored further. This would require a better understanding of the spin‐polarized charge injection processes and the development of suitable electrical contacts that can efficiently inject spin‐polarized carriers into the laser active region.
4. Nonlinear optics: In SHG applications, CHP films encounter several issues that hinder their efficiency. Phase mismatch, caused by film disorder, can lead to the cancellation of the SHG signal, reducing the overall conversion efficiency of light. To overcome this, integrating photonic crystals or metasurfaces with CHP films could enhance the local electromagnetic fields. Photonic crystals can be designed to have specific bandgap structures that can guide and manipulate light, while metasurfaces can provide sub‐wavelength control over the optical properties. By carefully engineering these structures, it may be possible to optimize the SHG process and increase the efficiency of CHP‐based SHG devices. Intense light exposure can also induce ion migration or structural decomposition in CHP films, affecting device stability. Understanding the underlying mechanisms of these processes and developing strategies to mitigate them is crucial. This could involve the use of stabilizing additives or the modification of the film composition to make it more resistant to light‐induced degradation.
5. Spintronics integration: In spintronics, the short spin lifetime in chiral ligand‐modified perovskites, typically in the picosecond range, is a major obstacle. Clarifying the microscopic mechanisms of the CISS effect, including the roles of SOC and chiral field effects, is essential. This requires a combination of theoretical and experimental studies, using advanced characterization techniques such as magnetic circular dichroism and spin‐resolved photoemission spectroscopy. Designing heterostructures that combine CHPs with topological insulators or 2D materials could extend the spin diffusion lengths, enabling the realization of spin transistors and logic gates. These heterostructures could provide a platform for studying and manipulating spin transport at the nanoscale.
6. Memory validation and sustainable design: CHP‐based FeRAM has shown great potential, but it requires comprehensive characterization beyond the basic ferroelectric properties. Write/erase endurance, retention, and switching speed are critical parameters that need to be thoroughly investigated. Establishing standard evaluation criteria, similar to those used for traditional FeRAM, would help in comparing the performance of different CHP‐based memory devices and in guiding the development of more reliable and efficient memory technologies. All‐organic CHPs offer a unique opportunity to eliminate lead contamination, which is a major concern in traditional perovskite materials. Leveraging these materials in the development of biocompatible wearable and implantable devices could open up new applications in the medical field. For example, CHP‐based memory devices could be used in implantable sensors for long‐term monitoring of physiological parameters. However, ensuring the biocompatibility of these materials and their long‐term stability in the human body requires further research. This could involve in vitro and in vivo studies to assess the interaction of CHPs with biological tissues and fluids.
7. System‐level integration and commercialization: Establishing industry standards for the characterization of CHPs is essential for their commercialization. Parameters such as CD, gCP‐EL, and spin lifetime need to be precisely defined and measured using standardized methods. This would enable consistent comparison of different CHP materials and devices, facilitating the development of a competitive market. Developing scalable manufacturing techniques is another key aspect of commercialization. Current fabrication methods for CHP‐based devices are often complex and time‐consuming, making them unsuitable for mass production. Exploring solution‐processable techniques, such as spin‐coating, inkjet printing, and spray‐coating, could offer more cost‐effective and efficient ways to produce CHP‐based devices. These techniques could be optimized to ensure high‐quality film formation and uniform device performance. Multimodal systems that merge CPL sensing, neuromorphic computing, and spintronics have the potential to drive progress in quantum optics and integrated photonic applications. However, achieving system‐level integration requires overcoming significant challenges, such as the compatibility of different components and the development of suitable interfaces. Interdisciplinary research efforts involving materials scientists, device engineers, and system designers will be crucial for the successful realization of these multimodal systems. By addressing these challenges, CHPs could transition from academic research to transformative technologies in various fields, including quantum computing, biomedical sensing, and energy‐efficient electronics.

## 6 Conclusion

CHPs have emerged as a promising class of materials at the intersection of chiroptics, optoelectronics, and spintronics. This review has explored CHP‐based devices across various architectures and applications, highlighting their unique properties. The inherent CD of CHPs makes them ideal for CPL sensing, while their tunable bandgaps, NLO properties, and spin‐related characteristics enable applications in diverse fields from neuromorphic computing to spintronics. All‐organic CHPs offer environmental and biocompatibility advantages. However, as outlined in the perspectives, significant challenges remain in material stability, device architecture, and system‐level integration. Overcoming these hurdles through interdisciplinary research will be key to realizing the full potential of CHPs in transformative technologies.

## Conflict of Interest

The authors declare no conflict of interest.
